# Systems Analysis of Biliary Atresia Through Integration of High-Throughput Biological Data

**DOI:** 10.3389/fphys.2020.00966

**Published:** 2020-08-07

**Authors:** Jun Min, Mylarappa Ningappa, Juhoon So, Donghun Shin, Rakesh Sindhi, Shankar Subramaniam

**Affiliations:** ^1^Department of Bioengineering, University of California, San Diego, La Jolla, CA, United States; ^2^Hillman Center for Pediatric Transplantation, Children’s Hospital of Pittsburgh of University of Pittsburgh Medical Center (UPMC), Pittsburgh, PA, United States; ^3^Department of Developmental Biology, McGowan Institute of Regenerative Medicine, University of Pittsburgh, Pittsburgh, PA, United States; ^4^Departments of Cellular and Molecular Medicine and Computer Science and Engineering, University of California, San Diego, La Jolla, CA, United States

**Keywords:** biliary atresia, systems biology, omics analysis, network reconstruction, integrative method

## Abstract

Biliary atresia (BA), blockage of the proper bile flow due to loss of extrahepatic bile ducts, is a rare, complex disease of the liver and the bile ducts with unknown etiology. Despite ongoing investigations to understand its complex pathogenesis, BA remains the most common cause of liver failure requiring liver transplantation in children. To elucidate underlying mechanisms, we analyzed the different types of high-throughput genomic and transcriptomic data collected from the blood and liver tissue samples of children suffering from BA. Through use of a novel integrative approach, we identified potential biomarkers and over-represented biological functions and pathways to derive a comprehensive network showing the dysfunctional mechanisms associated with BA. One of the pathways highlighted in the integrative network was hypoxia signaling. Perturbation with hypoxia inducible factor activator, dimethyloxalylglycine, induced the biliary defects of BA in a zebrafish model, serving as a validation for our studies. Our approach enables a systems-level understanding of human BA biology that is highlighted by the interaction between key biological functions such as fibrosis, inflammation, immunity, hypoxia, and development.

## Introduction

Biliary atresia (BA), or absence of extrahepatic ducts, which drain bile outside the liver, is a rare condition whose pathogenesis must be understood in order to limit its significant public health impact. Despite its rarity, BA is the most common cause of liver failure at birth and of liver transplantation in children, worldwide (Engelmann et al., 2007; Hsiao et al., 2008; Petersen et al., 2008). Disease pathogenesis is likely complex, based on circumstantial associations of BA with viral infections (Mack, 2007), a toxin-associated BA-like condition in a regional population of Australian sheep (Lorent et al., 2015), an interleukin 8 (IL8)-predominant inflammatory signature in BA-affected human liver tissues (Honsawek et al., 2005), and multiple susceptibility genes identified in genome-wide association studies in BA (Ningappa et al., 2015).

While the complex pathogenesis of BA requires critical evaluation, we face clinical and experimental challenges. Because the disease manifests at birth, investigating the role of various factors would require sequential evaluation of fetal and perinatal liver tissues, a task that poses ethical and technical challenges in humans. Furthermore, modeling the interaction between different potential etiologic factors in an animal model is also difficult in the presence of combinatorial effects of multiple susceptibility genes and unknown extraneous triggers required for disease onset and progression (Nakamura and Tanoue, 2013; Ningappa et al., 2015).

With the emergence of new observations that expand the established understanding of BA, a more comprehensive view is necessary to design therapeutics or preventative interventions. The most common ‘isolated’ form of BA in which disease is confined to the liver and bile ducts has been thought to result from inflammatory or acquired perinatal factors, while the less common ‘syndromic’ form of BA with laterality-type extrahepatic defects may result from defective genetic programs (Bates et al., 1998; Honsawek et al., 2005; Ningappa et al., 2015). However, both forms of BA are associated with poorly developed cilia in cholangiocytes, whose proper function is essential for the correct left-right placement of unpaired cardiovascular and gastrointestinal organs (Chu et al., 2012). Further, ‘isolated’ BA can also manifest with extrahepatic defects, albeit to a lesser degree and with lower incidence than ‘syndromic’ BA (Schwarz et al., 2013). These observations suggest that both forms of BA may share common mechanisms. Finally, despite the association of the ‘isolated’ BA with inflammation, bile duct patency after surgical reconstruction is not improved with adjunctive steroids that should limit inflammatory responses (Bezerra et al., 2014).

**FIGURE 1:**
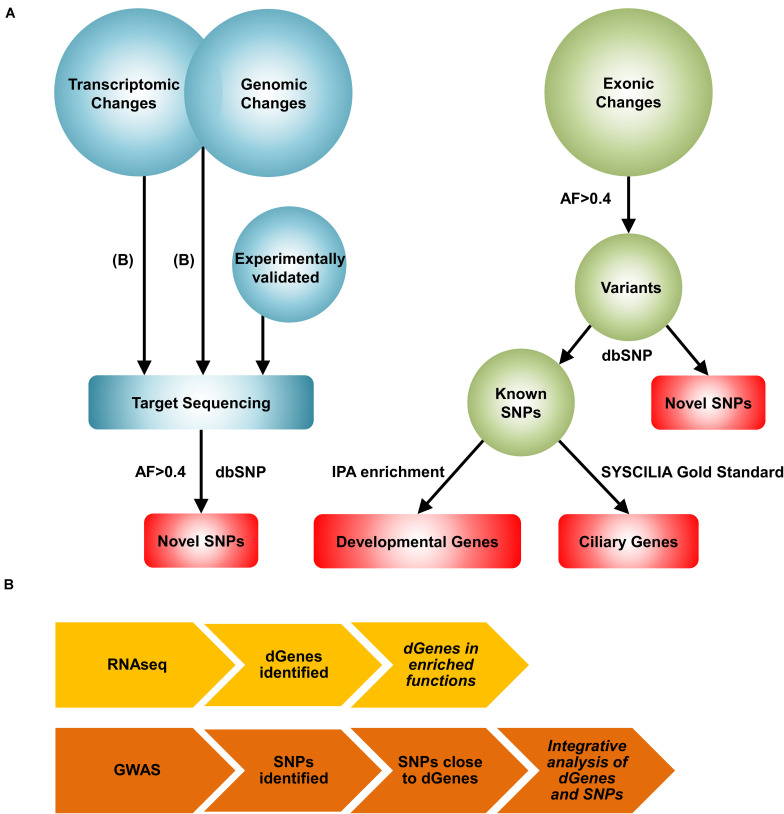
A novel systems biology approach for the reconstruction of the BA network. **(A)** Workflow of the Systems Biology approach. First, transcriptomic changes were analyzed from the RNAseq data to identify differentially regulated genes in the BA group. The list of differentially regulated genes were then used together with the list of significant genomic changes from the GWAS analysis to identify pairs of significant genes and SNPs. These results, along with experimentally validated genes, were further explored with target sequencing to identify highly common (AF>0.4 and AN>10) novel SNPs. Exomic changes were also examined with whole exome sequencing. From the whole exome data, the dbSNP 138 database was used to identify novel and known SNPs. Developmental genes mapped from the highly common known SNPs were identified using Ingenuity Pathway Analysis (IPA) enrichment. BA-related ciliary genes were identified with the SYSCILIA gold standard list. Red represents the genes and variants considered for the reconstruction of the BA network. AF: allele frequency, AN: total number of alleles. **(B)** The workflows for the transcriptomic and the integrative analyses are shown. For transcriptomic profiling, RNAseq analysis was performed to identify differentially regulated genes (dGenes). Then, enrichment analysis was performed to identify over-represented biological functions and pathways among dGenes. The integrative analysis involved selection of SNPs near dGenes from the GWAS data and application of set-based test in PLINK to identify pairs of significant genes and SNPs. Italicized results were target sequenced.

The clinical and experimental challenges and the existence of multiple forms of BA with different etiologies motivate us to create a comprehensive view of the disease through systems biology approaches. While numerous systems level approaches are available for analyzing specific types of omics data, there are no conventional network reconstruction methods available for integrating multiple types of omics data. We devised a novel, complex network reconstruction method that effectively integrates distinct high-throughput omics measurements (Figure 1). First, we performed a transcriptomic data analysis to find differentially regulated genes in the BA patients. Then, we performed an integrative analysis using transcriptomic and genomic data to identify pairs of differentially regulated genes and their nearby BA-associated single nucleotide polymorphisms (SNPs). Subsequently, we target-sequenced the genomic regions of significant genes identified from the previous analyses and the published literature to identify novel variants. Because, not all genomic changes lead to significant transcriptomic changes, we performed an unbiased screening of exonic regions through whole exome sequencing. Finally, we reconstructed a comprehensive BA network by integrating significant genes and variants to highlight relevant results and provide a mechanistic view of the complex pathogenesis of BA.

## Materials and Methods

### RNAseq Analysis

The pooled RNA libraries were prepared using Illumina’s mRNA Sequencing Sample Preparation kit (#RS-930-1001) from the explanted liver tissue samples of 6 BA patients and 6 normal pre-implant liver allografts. The pooled RNA libraries for the BA and the normal groups were sequenced on Genome Analyzer II with 36 bp paired-end at the Wistar Institute, Philadelphia. CASAVA software was used to generate a table of exon, gene, and splice counts in both BA and normal groups. DEseq, R package for RNAseq data, was used for differential analysis (Anders and Huber, 2010). The genes among the 40% lower quantile of the total read counts were removed to maximize the statistical power while maintaining the majority of the differentially regulated genes when the statistical analysis was performed without the removal. For variance estimation, the DEseq options of “blind” and “fit-only” were used. The resulting dispersion plot was checked for quality. The enrichment analysis of differentially regulated genes under the adjusted *p*-value cutoff of 0.1 using Benjamini-Hochberg method was performed using DAVID (Huang et al., 2009). EASE score, a modified Fisher exact *p*-value for DAVID enrichment, of 0.05 was used for the statistical cutoff.

### Integrative Analysis Using RNAseq and GWAS Data

An integrative pipeline was designed to identify differentially regulated genes that are accompanied by BA-associated SNPs. From the list of differentially regulated genes under the *p*-value of 0.05, SNPs that are within 20 kb upstream and downstream of each gene were selected as potential BA-associated SNPs. A set-based test from PLINK was performed to identify representative SNPs that are not in linkage disequilibrium (LD) with other SNPs for each gene (Purcell et al., 2007); we then performed a family based transmission disequilibrium test (TDT) for each SNP, calculated the average statistics for each gene based on the TDT statistics, and calculated the empirical *p*-value for each gene after permuting the phenotype labels of the dataset 10,000 times. The maximum number of representative SNPs for each gene was set to the default value of 5. For the GWAS TDT within the set-based test, 36 family trio genotypes on HumHap Infineum 550K Illumina SNP arrays were used; each family trio consisted of two unaffected parents and one affected child. TDT was preferred over the conventional chi-squared test with a principal component analysis (PCA) because the evidence of both linkage and association from TDT can increase the confidence of the biomarkers identified by the integrative results. Furthermore, for quality control of the GWAS data, SNPs with (i) more than 10% missing genotype across all individuals, (ii) minor allele frequency of less than 0.05, (iii) Hardy-Weinberg equilibrium of greater than 0.001, or (iv) Mendelian error rate of greater than 0.1 across all individuals were excluded from the test. In addition, Mendelian error rate of 0.05 was used to exclude families that did not meet the threshold. An empirical *p*-value cutoff of 0.05 was used for the set-based test.

### Target Sequencing Analysis

The DNA libraries were prepared from the blood of 43 BA patients using SureSelect kits from Agilent Technologies. A total of 24 genes and their 20 kb upstream and downstream sequences were captured using Haloplex custom probes from Agilent Technologies and sequenced in Illumina Hiseq2500 with 100 bp paired-end options. The majority of the genes that were target sequenced were potential BA-related genes derived from the results of RNAseq and integrative analyses; a few experimentally validated genes were included with this set. From the RNAseq results, CXCL5, CXCL10, SAA1, SAA2, ORM1, and IL8 were selected based on their significant up- and down-regulation in the enriched chemokine signaling and inflammatory pathways. From the integrative analysis using the set-based test, the following genes were selected based on their biological annotation and potential contribution to the pathogenesis of BA: EPHB2, vimentin (VIM), GDNF family receptor alpha 1 (GFRA1), DGAT2, FGF23, G protein-coupled receptor, class C, group 5, member A (GPRC5A), ANXA2, alanyl aminopeptidase (ANPEP), lipase, endothelial (LIPG), receptor (G protein-coupled) activity modifying protein 1 (RAMP1), LBP, C6, SLCO4C1, CFTR, and COL15A1. Lastly, the following genes were selected based on the published literature and the GWAS results: ARF6, mannosidase, alpha, class 1A, member 2 (MAN1A2), hypoxia inducible factor 1, alpha subunit (HIF1A), and hypoxia inducible factor 1, alpha subunit inhibitor (HIF1AN).

The alignment for the target sequencing data was performed using Burrows-Wheeler Aligner (BWA) against the hg19 human reference genome (Li and Durbin, 2009). Genome Analysis Toolkit (GATK) v3.3-0 was used for local realignment and variant calling with HaplotypeCaller (McKenna et al., 2010). The hard filters, QualByDepth (QD) <2.0, FisherStrand (FS) >60, RMSMappingQuality <40.0, HaplotypeScore >13.0, MappingQualityRankSumTest < -12.5, and ReadPosRankSumTest < -8, were used to remove potential false-positive SNPs. For indels, a similar filtering was applied. The filtered variants were then annotated using the dbSNP 138 database and the VCF annotation v1.0 from SnpEff. The variants were considered to be novel if they were not found in the dbSNP 138 database. The additional filters of AF>0.4 and AN>10 were applied to the annotated variants. The quality and the coverage of the sequencing data were checked with FastQC, Integrative Genomics Viewer (IGV), and CalculateHsMetrics from Picard (Thorvaldsdóttir et al., 2013).

### Whole Exome Sequencing Analysis

The exome libraries were prepared from the blood DNA of 54 BA patients using SureSelect XT Human All Exon V5 Library from Agilent Technologies and then sequenced in Illumina Hiseq2500 with 100 bp paired-end options. Similar to the target sequencing data analysis, BWA was used for alignment and GATK for variant calling and filtration. Potential PCR duplicates were identified and removed with PICARD. Base quality score recalibration from GATK was applied to the whole exome data. The quality and the coverage of the exome sequencing data were checked with FastQC, IGV, and CalculateHsMetrics from Picard. The highly common variants passing the filters of AF>0.4, AN>10, as well as having “moderate” or “high” variant effect from the VCF annotation were selected for further analyses. The “moderate” and “high” variant effects include variant functional categories such as missense, frameshift, splice acceptor, and stop-gained. The novel variants from the filtered list were annotated with the dbNSFP database for functional prediction of missense variants (Liu et al., 2011). In particular, SIFT, Polyphen2, and LRT from the dbNSFP database were used.

A list of the previously identified BA-associated SNPs was derived to help identify SNPs from the whole exome data that show significantly different genomic changes with respect to control. The list was derived from the results of several GWAS studies performed at the Children’s Hospital of Philadelphia and the Children’s Hospital of Pittsburgh. The total numbers of BA patients and normal patients across the multiple GWAS datasets were 74 and 1617, respectively. The common SNPs in both the list of GWAS SNPs and the filtered variants from the whole exome data were selected for enrichment analysis using the Ingenuity Pathway Analysis (IPA, QIAGEN Inc.); genes involved in ciliary development and function were identified using SYSCILIA gold standard (SCGSv1) (van Dam et al., 2013).

### Whole Exome Network Reconstruction

A custom human interaction network was created in Cytoscape (Shannon et al., 2003), a network visualization tool, by integrating protein-protein interaction data from BIOGRID version 3.3.122 (Stark et al., 2006), transcription factor interaction from TRANSFAC (Matys et al., 2003), and pathway information from KEGG pathways (Kanehisa and Goto, 2000). The topological features of this custom human interaction network, nodes and edges, were used to create the whole exome network for BA, along with the first neighbors of the genes mapped from the highly common variants from the whole exome data. The first neighbors are genes that interact directly with the target genes in a network with no intermediate genes between them. Then, BINGO Cytoscape plugin with default parameters was used to identify over-represented biological processes to create a GO network (Maere et al., 2005). The relevant biological processes that passed Benjamini-Hochberg-corrected *p*-value of 0.05 were included in the network.

### Biliary Atresia Network Reconstruction

The list of significant genes was derived from the combined results of high-throughput analyses: (i) all of the genes from the RNAseq and integrative analyses that were target sequenced, (ii) the genes mapped from the highly common novel SNPs (AF>0.4 and AN>10) from the whole exome data, (iii) BA-related ciliary genes mapped from the highly common known SNPs from the whole exome data, and (iv) the genes in the IPA-enriched biological categories from the whole exome data. The final list of significant genes was as follows: USP6, CXCL5, TNC, C6, VIM, KLRK1, ARF6, ANPEP, GPRC5A, CXCL10, EPHB2, CD44, HIF1AN, SAA1, SAA2, LBP, RAMP1, SLCO4C1, COL15A1, FGF23, MUC6, ANXA2, ORM1, NPC1, HIF1A, DGAT2, LIPG, NEUROD1, HHLA2, GFRA1, IGFBP1, IL8, MAN1A2, HTT, INVS, FSHD region gene 1 family member B (FRG1B), and T cell receptor beta constant 2 (TRBC2).

The initial BA network was then extended to include the first and the second neighbors of these significant genes within the custom human interaction network. The second neighbors are genes that interact indirectly with the target genes with only one intermediate gene between them. Then, a smaller network was reconstructed to include as many significant genes that are within the second neighbors of each other as possible, i.e., the interactions among significant genes was our main criteria for selecting the genes for the BA network.

A minimalistic approach was used to condense the BA network by excluding as many neighbor genes and their interactions as possible, while maintaining all the significant genes as essential information. Our focus in the minimalist approach was centered around molecular players (genes) that were either differentially expressed (in the RNA seq analysis) or had a polymorphism (in the whole exome or targeted exome analysis) or was identified in GWAS. We then use the Protein-Protein interaction network (BioGRID) to engender connections using minimal neighbors (most were first neighbor connections, with few exceptions, such as MAN1A2 to ARF6 (see Figure 5). When the connections involved too many intermediates, for e.g., HTT and VIM, the connections were ignored. Most interestingly, such a minimalist network, not only provided a biologically meaningful construct in identifying key players, but also yielded functional insights into mechanisms that are altered in the BA pathology.

After reconstruction of the BA network, each of the significant genes was annotated to identify common biological functions in the network. Furthermore, the common transcription factors that could potentially regulate the genes in the network were identified using DAVID’s UCSC_TFBS enrichment. EASE score, a modified Fisher exact *p*-value for DAVID enrichment, of 0.05 was used for the statistical cutoff.

### Zebrafish Validation Experiment

Experiments were performed with the approval of the Institutional Animal Care and Use Committee at the University of Pittsburgh. We used the following transgenic zebrafish lines: Tg(EPV.Tp1-Mmu.Hbb:EGFP)um14 [referred to here as Tg(Tp1:GFP)], Tg(EPV.Tp1-Mmu.Hbb:hist2h2l-mCherry)s939 [referred to here as Tg(Tp1:H2B-mCherry)], and Tg(EPV.Tp1-Mmu.Hbb:mCherry-CAAX)s733 [referred to here as Tg(Tp1:mCherry-CAAX)].

1000x stock solutions of dimethyloxaloylglycine (DMOG) (APExBIO, Houston, TX) were prepared in 100% DMSO and diluted to 100 μM with egg water. Larvae were treated with DMOG from 72 to 120 h post-fertilization (hpf). A 0.1% DMSO solution in egg water was used as a control.

PED6 assay was performed with 0.3 μg/ml of PED6 (Life Technologies, Grand Island, New York) for 3 h as previously described (Farber et al., 2001). BODIPY C5 assay was performed with 0.5 μM BODIPY C5 (Life Technologies, Grand Island, New York) for 2 h as previously described (Carten et al., 2011). Images for PED6 and BODIPY C5 assays were obtained using epifluorescence and confocal microscopes, respectively.

Whole-mount immunostaining was performed as previously described (Dong et al., 2007), using a rabbit polyclonal anti-Abcb11 (1:200; PC-064, Kamiya Biomedical, Seattle, WA) and Alexa Fluor 647-conjugated secondary antibodies (1:500; Life Technologies, Grand Island, NY).

Zeiss LSM700 confocal and Leica M205 FA epifluorescence microscopes were used to obtain image data. Confocal stacks were analyzed using the ZEN (black edition) software. The number of BECs and liver area were measured using the ImageJ software and were shown as means ± standard deviation (SD). Unpaired two-tailed Student’s *t*-test was used for statistical analysis; *P* < 0.05 was considered statistically significant.

## Results

### Enriched Inflammation and Chemokine Signaling Pathway in Transcriptome

To identify differentially regulated genes, we performed RNAseq analysis on 2 pools of explanted liver tissue samples from 6 BA patients and 6 normal pre-implant liver allografts. With DESeq package, we identified 98 significantly differentially regulated genes under the adjusted *p*-value cutoff of 0.1 using Benjamini-Hochberg method for multiple testing correction. To identify relevant biological pathways associated with these differentially regulated genes, we performed enrichment analysis with Database for Annotation, Visualization and Integrated Discovery (DAVID) online tool. This revealed several over-represented, or enriched, Kyoto Encyclopedia of Genes and Genomes (KEGG) pathways such as the chemokine signaling pathway (*p* = 2.0E-3), the toll-like receptor signaling pathway (*p* = 3.6E-2), and the cytokine-cytokine receptor interaction (*p* = 3.9E-2). It also revealed enriched Gene Ontology (GO) biological processes such as inflammatory response (*p* = 2.9E-5), locomotory behavior (*p* = 4.5E-5), chemotaxis (*p* = 5.5E-5), defense response (*p* = 8.8E-5), and immune response (*p* = 2.7E-4).

**FIGURE 2:**
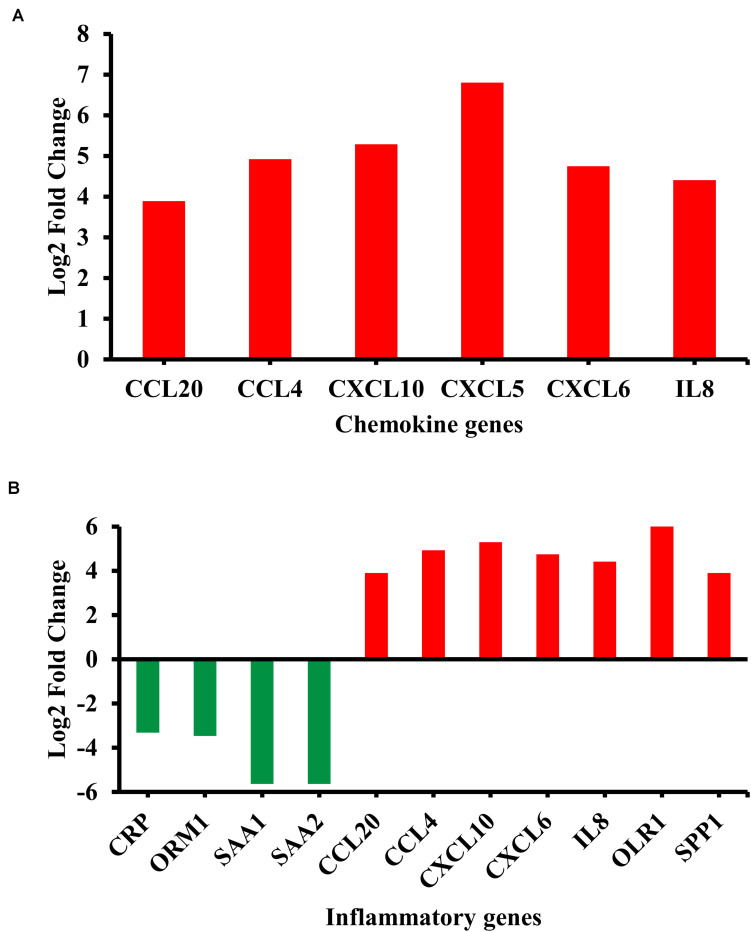
Differentially regulated genes in enriched biological categories. **(A)** Differentially regulated genes in the chemokine signaling pathway (*p* = 2.0E-3). **(B)** Differentially regulated genes in inflammatory response from Gene Ontology: Biological Process terms (*p* = 2.9E-5). Red indicates upregulation while green indicates downregulation. All genes passed the adjusted *p*-value cutoff of 0.1 using Benjamini-Hochberg method.

Among the enriched biological pathways and processes, the chemokine signaling pathway and inflammatory response were examined due to their biological relevance in the BA pathology (Jafri et al., 2009; Feldman and Mack, 2012; Bessho et al., 2014). Most of the genes in the chemokine signaling pathway, including chemokine (C-X-C motif) ligand 5 (CXCL5) and IL8, showed significant upregulation in their transcripts (Figure 2A). By contrast, inflammatory genes showed both up- and downregulation; many pro-inflammatory chemokines including IL8 were upregulated while some acute inflammatory genes, orosomucoid 1 (ORM1), serum amyloid A1 (SAA1), and serum amyloid A2 (SAA2), were downregulated in the BA group (Figure 2B).

### Significant Pairs of Genes and SNPs From the Integrative Analysis

We performed integrative analysis using the RNAseq and GWAS data to identify pairs of differentially regulated genes and their nearby BA-associated SNPs. Of the 500 differentially regulated genes from the RNAseq data under the *p*-value cutoff of 0.05, 10, 144 SNPs from 36 BA family trios that are within ∼20 kb up- and downstream of each gene were selected as potential BA-associated SNPs. We performed a set-based test in PLINK to identify 29 pairs of differentially regulated genes and their associated SNPs that passed the transmission disequilibrium test (TDT) (Table 1, Also see Supplementary Tables). These SNPs have significantly different allele frequency in the BA patients and can potentially regulate the expression of nearby genes. Among the 29 genes, complement component 6 (C6) and lipopolysaccharide binding protein (LBP) are involved in innate immunity while ephrin type-B receptor 2 (EPHB2) and annexin A2 (ANXA2) are associated with hepatic fibrosis (Kolgelier et al., 2015; Mimche et al., 2015; Zhou et al., 2016).

**TABLE 1:** Integrative analysis results from GWAS/RNAseq.

Gene	Empirical *P*-value	SNPs
SLCO4C1	0.0015	rs2600834
FGF23	0.0028	rs11063099, rs3812822, rs10437827
LIPG	0.0029	rs11664186
EPHB2	0.0042	rs6667416, rs4655107*, rs10753545, rs4655128, rs12027585
RAMP1	0.0066	rs10185142, rs1584243, rs6729271, rs6738488
GFRA1	0.0092	rs180552, rs180571, rs7087152, rs3901216, rs4751949
GPRC5A	0.0102	rs11055126
PID1	0.0102	rs13034774*, rs31276, rs7561470, rs883731, rs6724020
DGAT2	0.0122	rs1458836
EEF1A2	0.0126	rs11702306*
MME	0.0143	rs1025192, rs1816558
ANXA2	0.0144	rs4775260
MGP	0.0158	rs4762785
LBP	0.0160	rs2232618
AP1M2	0.0214	rs737337
SLC29A4	0.0230	rs6958502
VIM	0.0268	rs243013
C6	0.0298	rs11743598, rs3805715, rs1801033, rs751138
TESC	0.0328	rs10744888
COL15A1	0.0330	rs10819542, rs3780622, rs4743322
CD52	0.0332	rs12059495
C3orf25	0.0373	rs3138353
TMEM132A	0.0398	rs3794042
PCSK1N	0.0422	rs4824747, rs2280883
HOPX	0.0441	rs7684910*
ANPEP	0.0442	rs1439120
CFTR	0.0446	rs3808185, rs2237724
SLC28A1	0.0453	rs12438877, rs4247411*
PTHR1	0.0483	rs3729704, rs1531136

Results from the set-based test in PLINK using differentially regulated genes from the RNAseq data (*p* < 0.05) and GWAS SNPs that passed the TDT (*p* < 0.05) and are within 20 kb up- and downstream of differentially regulated genes. The empirical *p*-value cutoff was 0.05. A maximum of 5 SNPs representing each gene are listed. * denotes SNPs associated with transcription factor binding sites using SNP2TFBS web-based tool.

### Novel SNPs From the Target Sequencing Data

To identify novel variants around significant genes from the previous analyses, we sequenced the target regions of 24 selected genes from 43 BA patients. Most of these genes were selected based on the results from the RNAseq and integrative analyses. The final metrics for the target sequenced samples were 83.1% of bases showing at least 40 × coverage, which is enough to detect a significant number of variants. Most of the targeted genes were associated with at least one novel SNP passing the stringent allele frequency (AF) cutoff of 0.4 and the total number of alleles in called genotypes (AN) of greater than 10 (Table 2, also see Supplementary Tables). This stringent AF cutoff was applied to increase the confidence on potential biomarkers and their target genes. All of the highly common novel SNPs from the target sequencing data were non-coding suggesting that they may have indirect effects on the transcriptome or on clinical phenotypes.

**TABLE 2:** Number of highly common novel SNPs from target sequencing.

Gene	Number of novel SNPs (AF>0.4 and AN>10)
HIF1AN	6
FGF23	2
EPHB2	2
COL15A1	3
SLCO4C1	5
DGAT2	2
MAN1A2	2
ARF6	2
SAA1/2*	3
CXCL5	3
CXCL9	1
IL8	3
RAMP1	1
ANPEP	1
GFRA1	4
LIPG	1
LBP	2

Number of novel SNPs identified from target sequencing of 24 genes from 43 BA patients. The allele frequency (AF) cutoff and the total number of alleles in called genotypes (AN) cutoff were used to identify highly common SNPs. *SNPs were in close proximity to both SAA1 and SAA2.

### Novel Missense Variants From the Whole Exome Data

In addition to the targeted sequencing approach to discover new variants for the selected genes, we utilized the whole exome sequencing approach to discover novel variants from the unbiased exome of 54 BA patients. The whole exome sequencing analysis was also necessary because of the lack of non-coding variants discovered from the target sequencing. The final metrics cutoff for the whole exome sequencing was at least 80% of bases showing 30 × or higher coverage, which necessitated the removal of 5 BA samples from further analyses. We observed 51 novel variants that were highly common (AF>0.4 and AN>10) among the BA patients and can have potentially significant effect on their target genes according to the variant call format (VCF) annotation; 8 of these variants were missense mutations for 6 different genes (Table 3, Also see Supplementary Tables). Among the proteins encoded by these genes, ubiquitin carboxyl-terminal hydrolase 6 (USP6) regulates plasma membrane localization of ADP-ribosylation factor 6 (ARF6) (Martinu et al., 2004), while mucin 6, oligomeric mucus/gel-forming (MUC6) is involved in reactive biliary epithelium in viral hepatitis (Sasaki et al., 1998).

**TABLE 3:** Novel missense SNPs from whole exome sequencing.

Gene	Novel missense SNP (AF>0.4 & AN>10)			
	
	Genomic location	Allele frequency	Allele change	dbNSFP results
NPC1	chr18:21124945	0.653	C>G	Tolerated
TRBC2	chr07:142498833	0.500	A>T	NA
FRG1B	chr20:29632662	0.490	G>T	Tolerated
TRBV6-7	chr07:142143906	0.459	G>C	NA
	chr07:142143907	0.459	T>C	NA
	chr07:142144059	0.449	A>C	NA
USP6	chr17:5036210	0.459	T>G	Damaging*
MUC6	chr11:1017135	0.449	G>A	Damaging*

Novel missense SNPs identified from whole exome sequencing of 54 BA patients. The allele frequency (AF) cutoff and the total number of alleles in called genotypes (AN) cutoff were used to identify highly common SNPs. Out of 51 novel variants that were highly common among the BA patients, 8 variants were missense SNPs for 6 different genes. Functional prediction of these missense SNPs was interrogated using the dbNSFP database. *USP6 (“Damaging” from SIFT and LRT) and MUC6 (“Damaging” from Polyphen2).

### Significant Developmental Genes From the Whole Exome Data

We analyzed highly common known variants from the whole exome data to discover significant developmental genes and pathways. Since the whole exome of the normal group was not sequenced, and using other published whole exome data as control can significantly increase the error rate due to differences in experimental and bioinformatic protocols, we derived the internal list of known BA-associated SNPs from the set of previous GWAS data. This allowed us to identify highly common known variants that have shown statistical difference with respect to control. After mapping these common variants into genes, we performed enrichment analysis using IPA to identify over-represented biological processes.

The IPA enrichment analysis on 262 genes mapped from the common variants in the internal list and the whole exome data revealed significantly enriched embryonic development (*p* = 1.94E-2) and hematological system development and function (*p* = 3.10E-2). The genes in embryonic development were neuronal differentiation 1 (NEUROD1), huntingtin (HTT), and insulin-like growth factor binding protein 1 (IGFBP1), while the genes in hematological system development and function were HERV-H LTR-associating 2 (HHLA2), CD44, tenascin C (TNC), and killer cell lectin-like receptor subfamily K, member 1 (KLRK1). A similar result was found when the enrichment was performed on the first neighbors, genes directly interacting with 262 genes, in the custom human interaction network derived from protein-protein and transcription factor interactions.

Another biological process that is relevant for BA is ciliary development and function. 11 of 262 common genes were involved in ciliary development and function according to the SYSCILIA gold standard list; these genes were doublecortin domain containing 2 (DCDC2), dynein, axonemal, heavy chain 11 (DNAH11), HTT, intraflagellar transport 88 (IFT88), inversin (INVS), MDM1, pericentriolar material 1 (PCM1), SAS-6 centriolar assembly protein (SASS6), SCL/TAL1 interrupting locus (STIL), spectrin repeat containing, nuclear envelope 2 (SYNE2), and WD repeat domain 35 (WDR35).

### Whole Exome Network

**FIGURE 3:**
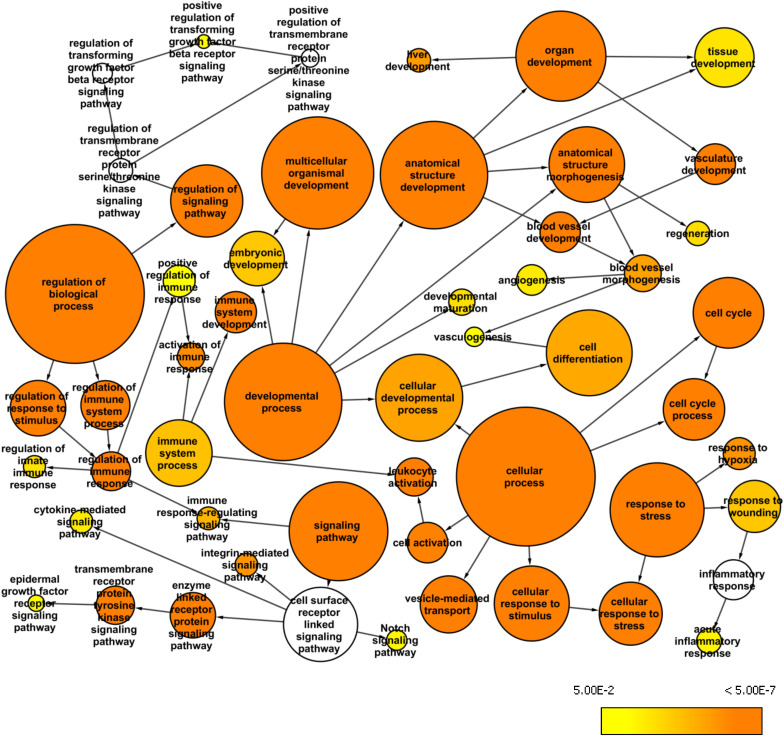
Whole exome network. This network was created in Cytoscape with the BINGO plugin to visualize over-represented Gene Ontology: Biological Processes among the genes mapped from the common variants from the whole exome data (AF>0.2 and AN>10) and their first neighbor genes within the custom human interaction network. The size of a node represents the number of genes annotated with that biological process while the color indicates different *p*-values for the significance of enrichment.

The whole exome data from the BA patients revealed a large number of variants that were excluded when we used the stringent AF cutoff of 0.4. Therefore, we used the AF cutoff of 0.2 to analyze the larger list of variants and to identify over-represented GO terms among the first neighbors of the mapped genes in the custom human interaction network (Figure 3). We also used BINGO Cytoscape plugin to create a network of enriched GO terms that may be relevant to BA pathology. Such identified GO terms were acute inflammatory response (*p* = 2.12E-03), vasculogenesis (*p* = 6.84E-03), embryonic development (*p* = 2.59E-05), liver development (*p* = 4.19E-07) regulation of immune response (*p* = 1.61E-03), epidermal growth factor receptor (EGFR) signaling pathway (*p* = 4.11E-03), and positive regulation of transforming growth factor beta (TGFβ) receptor signaling pathway (*p* = 5.92E-03). Among the enriched pathways, EGFR signaling can affect the development of BA through ARF6, while TGFβ receptor signaling can regulate fibrosis (Leask and Abraham, 2004).

### The Comprehensive Biliary Atresia Network

To effectively integrate the key results from different high-throughput data analyses, we utilized a novel systems biology approach to reconstruct a comprehensive BA network. Instead of deriving a large, complex network directly from the integrated genomic and transcriptomic data and applying a set of computational algorithms, we started with the list of significant BA genes and variants identified from different high-throughput data analyses (Figure 1). Using the custom human interaction network, we then derived the initial BA network by integrating the first and the second neighbors (0 or 1 intermediate genes) of the significant genes in a protein-protein interaction network. These neighbor genes show a close interaction with the significant genes at the protein level and would likely be affected during the pathogenesis of BA. Next, we selected significant genes that have first or second neighbor interaction with other significant genes in the network. The interaction among functionality significant genes was our main criteria for selecting the genes for the BA network under the assumption that the main components of a disease network would have close interactions with one another and represent altered function. After reconstructing the initial BA network, we used the minimalistic approach to reduce the number of neighbor genes to provide a highly condensed and interpretable network. A minimalistic approach was used to reconstruct the network to increase biological interpretability of the final networks and to help derive meaningful hypotheses for future experiments, while maintaining the relevant topology of the initial network.

**FIGURE 4:**
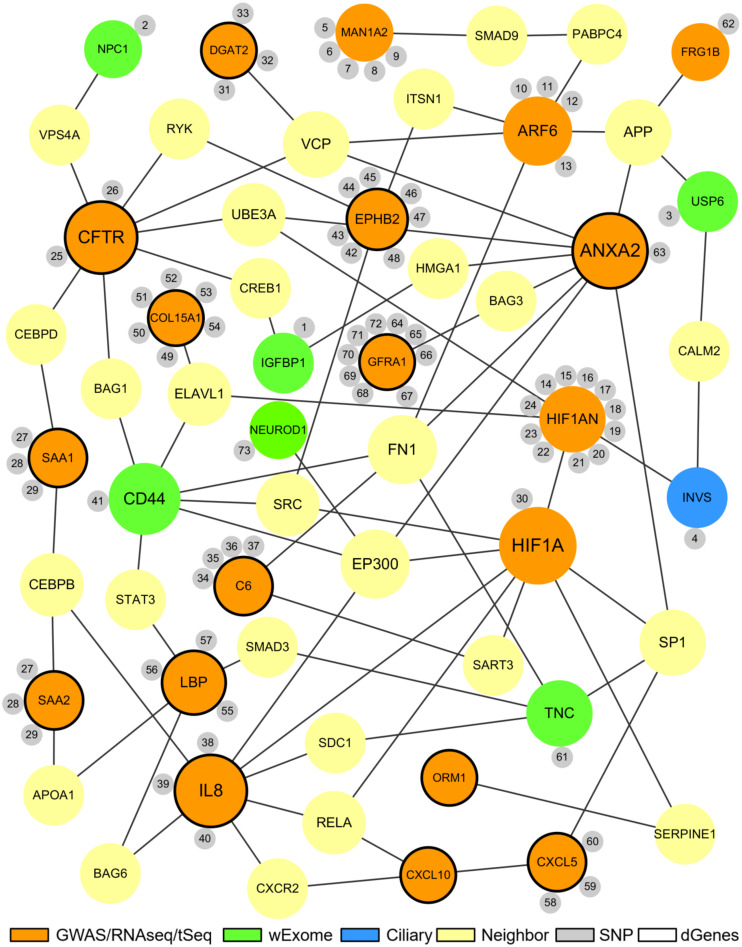
The proposed biliary atresia network. This network was created in Cytoscape by using the second neighbor interactions of the significant genes within the custom human interaction network. Interaction with other significant genes was the key criteria for selection into the final BA network. The size of a node depends on the connectivity within the network; the larger the node, the more connected it is to other genes. The orange nodes represent the significant genes derived from the GWAS, RNAseq, or target sequencing data while the light green nodes represent the significant genes from the whole exome data. One blue ciliary gene is also from the whole exome data. The yellow nodes represent the neighbor genes that link different significant genes through protein-protein interaction. The small gray nodes represent the SNPs that are associated with their attached genes. The entire list of SNPs, both novel and known, can be found in the Supplementary Tables. The black circular edges around the nodes represent differentially regulated genes (*p* < 0.05).

**FIGURE 5:**
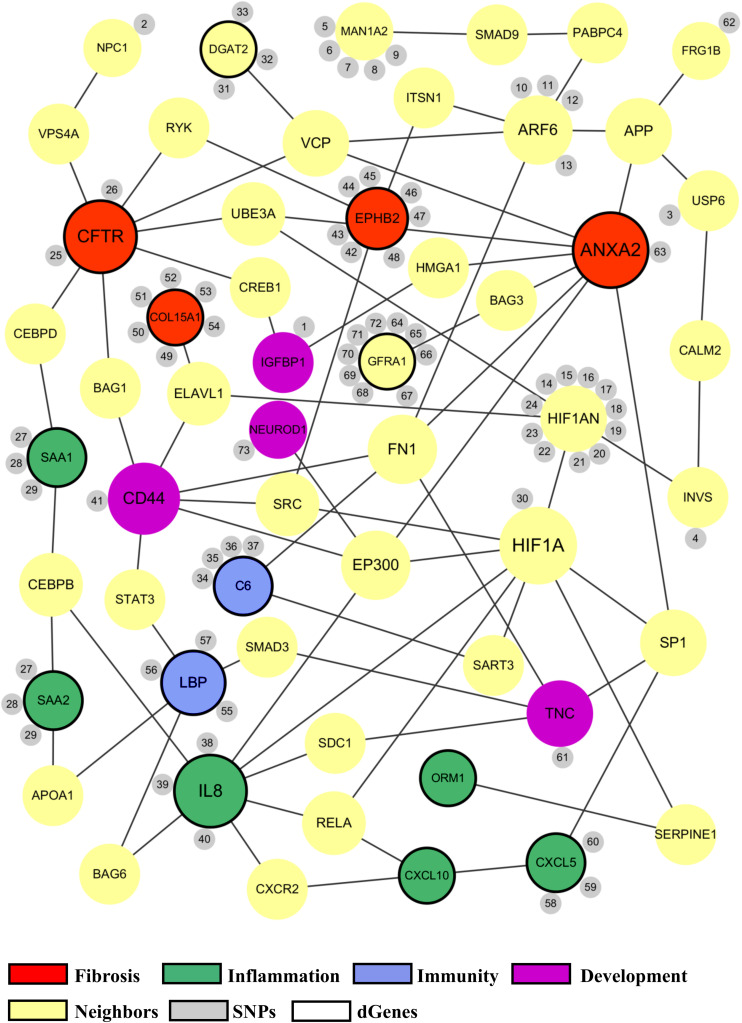
Common biological functions in the proposed biliary atresia network. All of the significant genes in the proposed BA network were annotated to identify common biological functions within the network. The red nodes represent the genes related to fibrosis, green related to inflammation, blue related to immune response, and purple related to development. The size of a node depends on the connectivity within the network; the larger the node, the more connected it is to other genes. The yellow nodes represent the neighbor genes that link different significant genes through protein-protein interaction. The small gray nodes represent the SNPs that are associated with their attached genes. The entire list of SNPs, both novel and known, can be found in the Supplementary Tables. The black circular edges around the nodes represent differentially regulated genes (*p* < 0.05).

The proposed BA network presents a comprehensive mechanistic view of the complex pathogenesis of BA (Figure 4). It highlights the sources and the interactions between the significant genes. The network has genes related to key biological functions associated with BA such as fibrosis, inflammation, immunity, and development (Figure 5). Lastly, the network also highlights the importance of hypoxia signaling, evidenced by HIF1A and HIF1AN with their numerous interactions and SNPs found from target sequencing.

We also discovered the common transcription factors that can regulate the genes in the final BA network using DAVID’s UCSC_TFBS enrichment (Also see Supplementary Tables); the top 5 transcription factors ranked by the *p*-values were ras responsive element binding protein 1 (RREB1), v-myb avian myeloblastosis viral oncogene homolog (MYB), forkhead box D3 (FOXD3), activator protein 1 (AP1), and hepatic leukemia factor (HLF).

### Zebrafish Validation of Hypoxia Inducible Factor Signaling

**FIGURE 6:**
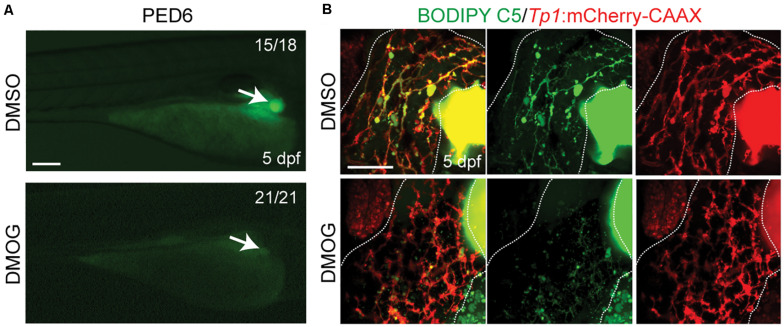
Activation of Hif1a signaling impairs biliary morphogenesis. **(A)** Epifluorescence images showing PED6 accumulation in the gallbladder (arrows). **(B)** Confocal projection images showing BODIPY C5 staining (green) and Tp1:mCherry-CAAX expression (red; BEC membrane) in the liver (dotted lines). Scale bars: 200 **(A)**, 50 μm **(B).**

**FIGURE 7:**
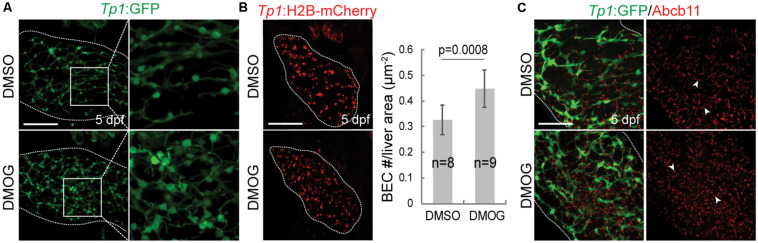
Activation of Hif1a signaling increases BEC number and impairs the proper formation of bile canaliculi. **(A)** Confocal projection images showing intrahepatic biliary network by Tp1:GFP expression in the liver (dotted lines) at 5 dpf. Boxed regions are enlarged. **(B)** Confocal projection images showing Tp1:H2B-mCherry expression (red; BEC nuclei) in the liver (dotted lines) at 5 dpf. Quantification of BEC number is shown. **(C)** Confocal projection images showing Abcb11 and Tp1:GFP expression in the liver (dotted lines) at 5 dpf. Arrowheads point to bile canaliculi. n indicates the number of larvae used for quantification. Scale bars: 50 μm; error bars: ± SD.

To validate the importance of key genes highlighted in the proposed BA network, we investigated the effect of hypoxia signaling triggered by HIF1A and HIF1AN on intrahepatic biliary network formation in a zebrafish model. The zebrafish model is often used to determine the role of genes or factors in the formation of the intrahepatic biliary network (Lorent et al., 2015; Ningappa et al., 2015), because the small size and transparency of zebrafish larvae allow for easy imaging of the entire liver, needed for visualizing the intricate intrahepatic biliary network. To visualize biliary network functionality, we first used a fluorescently labeled fatty acid reporter, PED6, which is metabolized into a component of bile in hepatocytes and accumulates in the gallbladder after biliary secretion (Farber et al., 2001). Zebrafish larvae treated with DMOG, which induces hypoxia signaling (van Rooijen et al., 2009), showed reduced PED6 accumulation in the gallbladder at 5 dpf (Figure 6A), suggesting a defect in the biliary network. Intrigued by this phenotype, we further used another fluorescently labeled lipid reporter, BODIPY C5, which reveals bile canaliculi and intrahepatic bile conduits as well as the gallbladder (Delous et al., 2012). The Tg(Tp1:mCherry-CAAX) line, which expresses a membrane-localized form of mCherry in biliary epithelial cells (BECs), was also used to visualize bile ductules. DMSO-treated larvae displayed BODIPY C5+ network co-localized with Tp1:mCherry-CAAX+ BECs, whereas DMOG-treated larvae did not display such network, indicating a defect in bile transport through bile ductules in the DMOG-treated larvae (Figure 6B). Intriguingly, DMOG-treated livers had denser biliary network (Figure 7A) and more BECs (Figure 7B) than DMSO-treated control livers, which were assessed using the Tg(Tp1:GFP) and Tg(Tp1:H2B-mCherry) lines that reveal the cytoplasm and nuclei of BECs, respectively. Bile canaliculi were shorter and more numerous in DMOG-treated livers (Figure 7C), as assessed by the expression of Abcb11, a bile salt export pump present in the bile canaliculi of hepatocytes.

## Discussion

The main findings of this paper are the identified potential biomarkers, the relevant biological functions and pathways, and the proposed BA network that was reconstructed using a novel integrative method using data from different measurements and analyses. Here, we delineate mechanisms involved in the complex pathogenesis of BA and the essential “interactions”, knowledge that is uniquely obtained from reconstruction of a highly condensed and interpretable network.

### The Complex Transcriptional Regulation of Inflammatory Genes

The transcriptomic data analysis revealed the enriched inflammatory and chemokine signaling pathways in the BA patients. The pro-inflammatory chemokines, IL8 and CXCL5, showed significant transcriptional upregulation, which is consistent with the inflammatory mechanism for the pathogenesis of the ‘isolated’ form of BA (Bessho and Bezerra, 2011). However, a few acute pro-inflammatory genes, SAA1, SAA2, and ORM1, were significantly downregulated. This suggests a more complex regulation of inflammatory genes in the pathogenesis of BA. Recently, the early clinical trial results from administrating corticosteroids, anti-inflammatory agents, to BA patients showed that suppression of inflammatory response may not always improve the condition of a patient (Bezerra et al., 2014). This finding, along with our results, demonstrate the need to further investigate the established inflammatory mechanism, which is perhaps complicated by the intricate balance and timing of its regulation during the various developmental stages of BA.

### The Roles of Fibrosis, Immunity, Bile Acid Transfer and Lipid Metabolism

The integrative analysis using the GWAS and the RNAseq data revealed many pairs of differentially regulated genes and SNPs that are involved in several different biological functions other than inflammation. For example, we identified EPHB2, ANXA2, collagen type XV alpha 1 (COL15A1), and cystic fibrosis transmembrane conductance regulator (CFTR) as fibrotic genes. EPHB2 and ANXA2 are related to hepatic fibrosis that is not only a prominent feature of BA but also a good predictor of outcome in mice following portoenterostomy (Farrington et al., 2010). In addition, COL15A1 is a potential marker for portal fibroblasts with a significant role in biliary fibrosis (Wells, 2014), while CFTR is a well-known gene for cystic fibrosis, a disease with similar symptoms as BA (Cant et al., 2014).

The integrative analysis revealed a few immune-related genes such as C6 and LBP. C6 is part of the complement system that is known for its involvement in inflammation and immunity against pathogens; C6 can help form a membrane attacking complex of the complement system that binds to the surface of bacterial cells and lyse them, while LBP helps protect against bacterial infections by facilitating acute-phase immunologic response (Nangaku et al., 1999; Schroedl et al., 2001; Ngure et al., 2009; Ricklin et al., 2010). These immune genes support the infection theory, a suggested etiology for BA.

Other biological functions, such as bile acid transfer and lipid metabolism, may also play significant roles in BA pathology. Solute carrier organic anion transporter family, member 4C1 (SLCO4C1) is part of the organic anion transporter family that is involved in the membrane transport of bile acid (Mikkaichi et al., 2004). This particular gene was significantly downregulated along with 6 nearby variants. Another significant gene from the transcriptomic profiling, diacylglycerol O-acyltransferase 2 (DGAT2), is involved in the production and accumulation of triglycerides in tissues and has been linked with insulin resistance and diabetes (Levin et al., 2007). Furthermore, we discovered a novel missense variant within Niemann-Pick disease, type C1 (NPC1) that is involved in the hedgehog signaling pathway and intracellular cholesterol transfer (Eaton, 2008). Defects in this gene can cause Niemann-Pick type C disease, a rare and incurable lipid storage disease.

### Biliary Atresia as a Developmental Disease

From the whole exome data, we observed the evidence of BA as a developmental disease. The enrichment result revealed the potential importance of embryonic development and hematological system development and function. This is consistent with the suggested pathogenic mechanism for the ‘syndromic’ form of BA- that multiple genomic changes could cause a problem during embryonic development of the liver and the bile ducts. This theory is further supported by the presence of situs inversus, which is an indication of dysregulated left-right axis determination during the embryonic development, in many BA patients (Bessho and Bezerra, 2011). One of the genes involved in left-right axis determination and ciliary development is INVS, whose role in BA pathology has been studied using experimental mouse models (Shimadera et al., 2007). Another gene involved in left-right axis determination is fibronectin 1 (FN1). Previous research has shown that FN1-null embryos had abnormal LR patterning and the pointed pole of the ventral node facing posteriorly instead of anteriorly (Pulina et al., 2014). Although we did not find conclusive evidence for FN1, it interacts with 4 other significant genes, C6, ARF6, ANXA2, and CD44, in the proposed final BA network. Therefore, FN1’s interaction with these proteins may qualify FN1 as a key protein in the BA network.

Cilia are microtubule-based organelles that are intricately involved in the left-right asymmetry process (Hirokawa et al., 2009). The enrichment analysis on the transcriptomic data revealed locomotory behavior, a biological process that is related to ciliary function. Furthermore, 11 genes from the whole exome data were related to ciliary development and function. Besides INVS, PCM1 is another ciliary gene that can interact with HTT, polo-like kinase 1 (PLK1), and centrosomal protein 290 (CEP290) to facilitate proper ciliary development and disassembly (Wang et al., 2013, 1). Although not in the SYSCILIA gold standard list of ciliary genes, fibroblast growth factor 23 (FGF23) can also affect ciliary function, because it belongs to the FGF family that can regulate cilia-driven nodal flow during the left-right axis specification (Hirokawa et al., 2009). FGF23 may also play a role in facilitating proper ciliary development by interacting with other known FGF proteins, such as FGF8.

### “Interaction” Knowledge From the Proposed BA Network

To evaluate BA as a complex disease with multiple mechanisms, we developed a BA network using the interactions of the significant genes discovered from different analyses. The proposed network provides analytic insights into how groups of genes with either similar or different biological functions can interact to affect the complex pathogenesis of BA. An example of interaction knowledge can be found in CFTR and its interactions. CFTR is the second most well-connected node in the network, being connected to 7 other significant genes through potential neighbor interactions, and it is also part of the fibrotic subnetwork with EPHB2, ANXA2, and COL15A1. From amongst the protein-protein interactions in the network, CFTR may influence acute inflammation represented by SAA1, embryonic development represented by IGFBP1, and hematological system development represented by CD44.

Another significant gene with numerous interactions in the network is IL8, a well-known inflammatory gene that is a part of the inflammation subnetwork with CXCL10, CXCL5, ORM1, SAA1, and SAA2. This gene is located at the center of the inflammation subnetwork and is well-connected to the other inflammatory genes, which suggests that IL8 may be a key regulator of inflammation in BA. Additionally, it is connected to LBP, one of the two genes involved in regulation of immune response that is closely related to inflammation.

A small subnetwork of genes representing the developmental mechanism of BA is present in the network. For example, CD44 is known for its wide variety of functions including but not limited to lymphocyte activation, recirculation, hematopoiesis, and tumor metastasis (Zhou et al., 2011). It is part of the developmental subnetwork from the whole exome data and is connected to another developmental gene, TNC. CD44 is also connected to 3 other functional subnetworks, namely, inflammation (IL8), immunity (LBP) and fibrosis (CFTR, COL15A1, and ANXA2). CD44 may be considered a key gene involved in the pathogenesis of BA serving as a mediator between different biological functions. Although not included in the network, HTT is another developmental gene that is well connected to many of the significant genes in the network.

In addition to the groups of genes with related biological functions, some of the individual genes and their connections warrant further analysis. For instance, USP6 is a gene involved in membrane localization of ARF6 and regulation of ARF6-dependent endocytic protein trafficking (Martinu et al., 2004). A recent study showed that ARF6 is involved in the EGFR pathway and is critical for proper bile duct formation, which suggests a non-superfluous role for USP6 in biliary atresia (Ningappa et al., 2015). According to the bioinformatics tools that can predict whether a missense variant can affect the protein function, namely SIFT and LRT, the novel variant at chr17:5036210 within USP6 is considered to be “damaging.” Furthermore, USP6 is connected to INVS through calmodulin 2 (CALM2), which suggests a potential interaction between the ARF6-dependent BA pathway and ciliary development.

### Common Transcription Factors in the BA Network

The proposed BA network with interaction knowledge also allows for the identification of common transcription factors that could interact with many of the genes in the network. One such factor is FOXD3, a transcription factor that participates in liver and lung formation from foregut endoderm (Guo et al., 2002, 3). A study showed that FOXD3 could activate osteopontin enhancer that is expressed in totipotent embryonic stem cells, which suggests that FOXD3 is a key transcriptional factor involved in managing the developmental aspect of the complex pathogenesis of BA (Guo et al., 2002, 3). Another important transcription factor is MYB that facilitates proper development of hematopoiesis during embryonic development and can be related to the complement system and coagulation cascade (Sandberg et al., 2005). Lastly, AP1 can regulate gene expression in response to a variety of stimuli, including cytokines, growth factors, stress, and bacterial and viral infections (Hess et al., 2004) thus implicating it in the regulation of the expression of inflammatory genes for the ‘syndromic’ form of BA.

### Potential BA Genes Not in the Network

Despite the valuable information provided by the network, we omitted some of the identified genes from high-throughput analyses due to their lack of interconnectivity with other significant genes in a previously known protein interaction network. These genes may still be significant because the proposed BA network represents only one large group of variants and genes that are highly interconnected; other groups of variants and genes, such as MUC6 and ANXA2, likely exist. MUC6, in particular, has been linked with viral hepatitis in biliary epithelium and has a highly common novel missense variant that can significantly damage the protein function (Sasaki et al., 1998). ANXA2 is another potentially relevant gene that was significantly upregulated in cholangiocytes in primary biliary cirrhosis from a recent study (Kido et al., 2009).

The proposed BA network shows significant involvement of HIF1A and HIF1AN with numerous connections to other genes as well as the high number of SNPs found from target sequencing. To validate the importance of these two genes in biliary network formation from the integrative network analysis, we investigated whether inducing hypoxia signaling with DMOG, which suppresses the proteasome-mediated degradation of HIF transcription factors, would cause biliary dysgenesis in a zebrafish model.

We first observed reduced PED6 accumulation in DMOG-treated zebrafish larvae livers at 5 dpf, which suggest problems with intestinal lipid absorption, biliary secretion, and biliary morphology (Farber et al., 2001; Matthews et al., 2004). Together with the defect in bile transport with BODIPY-C5 labeling and denser biliary network with more BECs from confocal images, phenotypic observations revealed negative effects of hypoxia signaling on the intrahepatic biliary network formation.

The validation of DMOG-induced hypoxia signaling in zebrafish is noteworthy. The loss of PED6 staining in the gall bladder despite increased numbers of biliary epithelial cells and a dense biliary network in the liver can only be explained by poor bile transport through shorter and presumably dysfunctional bile canaliculi. These findings also contrast with previous studies which induced sparse biliary networks in the liver after knockdown of GWAS-based susceptibility genes, and more closely recapitulate the “bile duct proliferation” seen in human biliary atresia.

## Conclusion

The proposed BA network has several advantages over existing postulated mechanisms: (i) the network can be used to study both the individual interactions among specific genes and the complex relationship among the major hubs of genes involved in fibrosis, immunity, inflammation, hypoxia, and development; (ii) the network is highly trainable; future experiments could refine the genes and their individual interactions from the existing version of the network; (iii) the network contains the variants with high allele frequency, which ensures a high degree of confidence and replicability; (iv) common biological functions, such as inflammation, regulation of immune response, and embryonic development, were shared between the proposed BA network and the whole exome network.

Due to the nature of studying a rare population such as BA, a limitation of our results is the lack of a large sample size providing higher statistical power. We attempted to alleviate this problem by integrating the results of different datasets as well as applying a stringent allele frequency cutoff to only select the variants that are highly frequent in the BA patients. Furthermore, the lower statistical power is mitigated by the consistency of our findings with the published results. Many of the genes and variants identified from the various analyses and our constructed BA network were involved in the known biological functions of BA, while a few significant genes in the proposed BA network were validated from the published literature.

This study shows the use of a systems biology approach in understanding how BA-related genes, SNPs, and their associated biological functions and pathways identified from different high-throughput genomic and transcriptomic data can be related in normal and pathological development. The study provides a mechanistic view of the complex pathogenesis of BA through a novel network reconstruction method which reveals key insights into how significant genes with different biological functions interact to contribute to the development of BA. Future *in vitro* and *in vivo* experiments testing each perturbation within the proposed BA network are possible and can provide valuable insights, as demonstrated by this study’s validation experiment with the zebrafish model on the impact of hypoxia signaling on the intrahepatic biliary network formation. For developmental genes, the timing of their regulation and the resulting phenotypic changes can be investigated in animal models. In addition, it is possible to identify a group of significant genes and variants from the network that can explain the differences between rejection and non-rejection groups of BA liver-transplant patients. Finally, this study demonstrates the potential benefit of integrating different high-throughput data to reconstruct a disease network that can provide a comprehensive understanding of the disease and yield essential biological insights.

## Data Availability Statement

The datasets generated for this study can be found in the GSE136270, GEO Database, embargoed for 6 months. We have requested the data to be available to reviewers.

## Ethics Statement

Experiments were performed with the approval of the Institutional Animal Care and Use Committee 204 at the University of Pittsburgh.

## Author Contributions

JM contributed to the analysis and systems biology interpretation and an initial draft of the manuscript. MN, JS, and DS contributed to various aspects of experimental measurements and zebrafish validation studies. RS was initiated the project. RS and SS made designed the project and supervised the research and revision process. All authors approved the final version of the manuscript.

## Conflict of Interest

The authors declare that the research was conducted in the absence of any commercial or financial relationships that could be construed as a potential conflict of interest.
