# Supplementary material for: Systems Analysis of Biliary Atresia Through Integration of High-Throughput Biological Data
Source: Front Physiol. 2020 Aug 7;11:966. doi: 10.3389/fphys.2020.00966 (PMC7426509; doi:10.3389/fphys.2020.00966)
Supplement: Supplementary file 1 [file Table_1.DOCX]

Supplementary Material

Systems analysis of biliary atresia through integration of high-throughput biological data

**Supplementary Table 1. Highly common novel variants from target sequencing**

| **Gene** | **Novel SNPs (AF>0.4 & AN>10)** | | |
| --- | --- | --- | --- |
|  | **Genomic location** | **Allele frequency** | **Allele change** |
| HIF1AN | chr10:102371717 | 0.558 | A > G |
|  | chr10:102381938 | 0.430 | G > C |
|  | chr10:102335587 | 0.419 | C > A |
|  | chr10:102335588 | 0.419 | C > T |
|  | chr10:102371719 | 0.452 | A > G |
|  | chr10:102288649 | 0.417 | T > A |
| FGF23 | chr12:4445620 | 0.465 | A > G |
|  | chr12:4445618 | 0.547 | A > G |
| EPHB2 | chr01:23245302 | 0.488 | G > A |
|  | chr01:23224633 | 0.500 | G > A |
| COL15A1 | chr09:101791486 | 0.477 | A > G |
|  | chr09:101838582 | 0.477 | T > A |
|  | chr09:101802438 | 0.474 | G > A |
| SLCO4C1 | chr05:101598264 | 0.453 | A >C |
|  | chr05:101605510 | 0.442 | T > C |
|  | chr05:101605514 | 0.442 | G > A |
|  | chr05:101605515 | 0.442 | T > G |
|  | chr05:101627933 | 0.462 | T > C |
| DGAT2 | chr11:75511683 | 0.476 | A > G |
|  | chr11:75510671 | 0.405 | A > T |
| MAN1A2 | chr01:117991089 | 0.419 | T > C |
|  | chr01:117991086 | 0.571 | T > G |
| ARF6 | chr14:50369743 | 0.462 | G > T |
|  | chr14:50369741 | 0.480 | C > T |
| SAA1/2* | chr11:18306706 | 0.479 | G > A |
|  | chr11:18275399 | 0.465 | G > A |
|  | chr11:18244418 | 0.659 | T > G |
| CXCL5 | chr04:74837722 | 0.500 | A > G |
|  | chr04:74837726 | 0.500 | C > G |
|  | chr04:74853963 | 0.453 | A > C |
| CXCL9 | chr04:76928543 | 0.452 | C > A |
| IL8 | chr04:74617686 | 0.541 | T > G |
|  | chr04:74612590 | 0.444 | T > A |
|  | chr04:74612589 | 0.522 | C > G |
| RAMP1 | chr02:238760567 | 0.429 | G > T |
| ANPEP | chr15:90311139 | 0.460 | T > A |
| GFRA1 | chr10:118013852 | 0.429 | G > A |
|  | chr10:118013856 | 0.429 | G > A |
|  | chr10:118013848 | 0.500 | G > A |
|  | chr10:118013877 | 0.500 | G > A |
| LIPG | chr18:47090001 | 0.682 | T > A |
| LBP | chr20:36950014 | 0.574 | T > A |

**Supplementary Table 2. Identity of the numbered SNPs in the proposed biliary atresia network**

| **#** | **SNP** | **Gene** |
| --- | --- | --- |
| 1 | rs4619 | IGFBP1 |
| 2 | chr18:21124945:C->G | NPC1 |
| 3 | chr17:5036210:T->G | USP6 |
| 4 | rs3813712 | INVS |
| 5 | rs12131109 | MAN1A2 |
| 6 | rs7531715 | MAN1A2 |
| 7 | rs6657965 | MAN1A2 |
| 8 | chr1:117991089:T->C | MAN1A2 |
| 9 | chr1:117991086:T->G | MAN1A2 |
| 10 | rs3126184 | ARF6 |
| 11 | rs10140366 | ARF6 |
| 12 | chr14:50369743:G->T | ARF6 |
| 13 | chr14:50369741:C->T | ARF6 |
| 14 | rs2495725 | HIF1AN |
| 15 | rs7092999 | HIF1AN |
| 16 | rs3763696 | HIF1AN |
| 17 | rs2495718 | HIF1AN |
| 18 | rs1110286 | HIF1AN |
| 19 | chr10:102371717:A->G | HIF1AN |
| 20 | chr10:102381938:G->C | HIF1AN |
| 21 | chr10:102335587:C->A | HIF1AN |
| 22 | chr10:102335588:C->T | HIF1AN |
| 23 | chr10:102371719:A->G | HIF1AN |
| 24 | chr10:102288649:T->A | HIF1AN |
| 25 | rs3808185 | CFTR |
| 26 | rs2237724 | CFTR |
| 27 | chr11:18275399:G->A | SAA1/2 |
| 28 | chr11:18306706:G->A | SAA1/2 |
| 29 | chr11:18244418:T->G | SAA1/2 |
| 30 | rs1957757 | HIF1A |
| 31 | rs1458836 | DGAT2 |
| 32 | chr11:75511683:A->G | DGAT2 |
| 33 | chr11:75510671:A->T | DGAT2 |
| 34 | rs11743598 | C6 |
| 35 | rs3805715 | C6 |
| 36 | rs1801033 | C6 |
| 37 | rs751138 | C6 |
| 38 | chr4:74617686:T->G | IL8 |
| 39 | chr4:74612590:T->A | IL8 |
| 40 | chr4:74612589:C->G | IL8 |
| 41 | rs9666607 | CD44 |
| 42 | rs6667416 | EPHB2 |
| 43 | rs4655107 | EPHB2 |
| 44 | rs10753545 | EPHB2 |
| 45 | rs4655128 | EPHB2 |
| 46 | rs12027585 | EPHB2 |
| 47 | chr1:23245302:G->A | EPHB2 |
| 48 | chr1:23224633:G->A | EPHB2 |
| 49 | rs10819542 | COL15A1 |
| 50 | rs3780622 | COL15A1 |
| 51 | rs4743322 | COL15A1 |
| 52 | chr9:101791486:A->G | COL15A1 |
| 53 | chr9:101838582:T->A | COL15A1 |
| 54 | chr9:101802438:G->A | COL15A1 |
| 55 | rs2232618 | LBP |
| 56 | chr20:36980457:G->A | LBP |
| 57 | chr20:36950014:T->A | LBP |
| 58 | chr4:74853963:A->C | CXCL5 |
| 59 | chr4:74837722:A->G | CXCL5 |
| 60 | chr4:74837726:C->G | CXCL5 |
| 61 | rs1757095 | TNC |
| 62 | chr20:29632662:G->T | FRG1B |
| 63 | rs4775260 | ANXA2 |
| 64 | rs180552 | GFRA1 |
| 65 | rs180571 | GFRA1 |
| 66 | rs7087152 | GFRA1 |
| 67 | rs3901216 | GFRA1 |
| 68 | rs4751949 | GFRA1 |
| 69 | chr10:118013852:G->A | GFRA1 |
| 70 | chr10:118013856:G->A | GFRA1 |
| 71 | chr10:118013848:G->A | GFRA1 |
| 72 | chr10:118013877:G->A | GFRA1 |
| 73 | rs1801262 | NEUROD1 |

*The SNPs connected to SAA1 and SAA2 are shared since they are within close proximity to both genes but not in the coding region of either gene.

**Supplementary Table 3. Common transcription factors for the genes in the network**

| **Term** | **Count** | **%*** | **P-value** | **Genes** |
| --- | --- | --- | --- | --- |
| RREB1 | 30 | 55.56 | 0.00 | UBE3A, PABPC4, SART3, ITSN1, SRC, EPHB2, APP, HIF1AN, SERPINE1, VPS4A, FN1, SMAD9, RYK, RELA, SMAD3, CFTR, HMGA1, STAT3, ANXA2, ORM1, SDC1, NPC1, HIF1A, EP300, SP1, VCP, DGAT2, NEUROD1, GFRA1, CALM2 |
| MYB | 32 | 59.26 | 0.00 | UBE3A, C6, PABPC4, ARF6, SART3, ITSN1, SRC, EPHB2, APP, BAG1, CD44, BAG3, VPS4A, FN1, SMAD9, MAN1A2, RYK, CREB1, COL15A1, SMAD3, ELAVL1, CFTR, STAT3, ANXA2, SDC1, HIF1A, SP1, VCP, INVS, GFRA1, IGFBP1, CALM2 |
| FOXD3 | 26 | 48.15 | 0.00 | CXCL5, UBE3A, C6, ITSN1, EPHB2, APP, CD44, SERPINE1, VPS4A, FN1, SMAD9, MAN1A2, RYK, CREB1, COL15A1, ELAVL1, SMAD3, CFTR, STAT3, ANXA2, SDC1, NPC1, HIF1A, SP1, GFRA1, CALM2 |
| AP1 | 38 | 70.37 | 0.00 | USP6, UBE3A, PABPC4, ARF6, SRC, EPHB2, APP, APOA1, CD44, HIF1AN, BAG3, SERPINE1, VPS4A, FN1, CEBPB, SMAD9, MAN1A2, IL8, RYK, CREB1, RELA, COL15A1, SMAD3, CFTR, HMGA1, STAT3, ANXA2, SDC1, NPC1, HIF1A, EP300, SP1, VCP, INVS, DGAT2, NEUROD1, GFRA1, CALM2 |
| HLF | 28 | 51.85 | 0.01 | USP6, UBE3A, C6, SART3, ITSN1, APP, BAG1, HIF1AN, SAA2, SAA1, SERPINE1, FN1, MAN1A2, COL15A1, SMAD3, ELAVL1, FRG1B, CFTR, HMGA1, ANXA2, ORM1, SDC1, HIF1A, INVS, NEUROD1, GFRA1, IGFBP1, CALM2 |
| RFX1 | 38 | 70.37 | 0.01 | USP6, CXCL5, C6, PABPC4, ARF6, SART3, ITSN1, SRC, CXCL10, EPHB2, APP, CD44, BAG1, BAG3, SERPINE1, VPS4A, LBP, FN1, CEBPB, MAN1A2, CEBPD, CREB1, RELA, COL15A1, SMAD3, HMGA1, STAT3, SDC1, NPC1, HIF1A, EP300, SP1, VCP, INVS, DGAT2, NEUROD1, GFRA1, CALM2 |
| IRF7 | 30 | 55.56 | 0.01 | CXCL5, UBE3A, C6, PABPC4, ITSN1, CXCL10, EPHB2, APP, CD44, BAG1, HIF1AN, BAG3, FN1, COL15A1, SMAD3, ELAVL1, FRG1B, HMGA1, STAT3, ANXA2, SDC1, NPC1, HIF1A, EP300, SP1, INVS, GFRA1, NEUROD1, IGFBP1, CALM2 |
| CREBP1 | 31 | 57.41 | 0.01 | USP6, CXCL5, UBE3A, PABPC4, ITSN1, SART3, EPHB2, APP, CD44, BAG1, HIF1AN, VPS4A, FN1, CREB1, RELA, COL15A1, SMAD3, ELAVL1, CFTR, STAT3, ANXA2, SDC1, NPC1, HIF1A, EP300, SP1, INVS, NEUROD1, GFRA1, IGFBP1, CALM2 |
| PAX6 | 34 | 62.96 | 0.01 | USP6, UBE3A, C6, ITSN1, EPHB2, APP, CD44, BAG1, HIF1AN, SAA1, BAG3, SERPINE1, FN1, CEBPB, SMAD9, MAN1A2, RYK, CREB1, RELA, SMAD3, ELAVL1, CFTR, HMGA1, ANXA2, SDC1, NPC1, HIF1A, EP300, SP1, VCP, INVS, NEUROD1, GFRA1, CALM2 |
| SRY | 28 | 51.85 | 0.01 | UBE3A, C6, PABPC4, ITSN1, EPHB2, APP, CD44, BAG3, SERPINE1, LBP, FN1, SMAD9, MAN1A2, RYK, CREB1, COL15A1, SMAD3, ELAVL1, CFTR, FRG1B, STAT3, ANXA2, HIF1A, EP300, INVS, SP1, GFRA1, CALM2 |
| CDC5 | 29 | 53.70 | 0.02 | UBE3A, C6, PABPC4, ARF6, CXCR2, ITSN1, SRC, EPHB2, CXCL10, APP, SAA2, CD44, SAA1, VPS4A, FN1, MAN1A2, CREB1, SMAD3, ELAVL1, CFTR, HMGA1, ANXA2, SDC1, HIF1A, EP300, DGAT2, SP1, NEUROD1, GFRA1 |
| NCX | 29 | 53.70 | 0.02 | USP6, UBE3A, ITSN1, SART3, EPHB2, APP, SAA2, CD44, SAA1, SERPINE1, FN1, MAN1A2, RYK, CREB1, RELA, COL15A1, SMAD3, ELAVL1, CFTR, STAT3, ANXA2, ORM1, SDC1, EP300, SP1, INVS, DGAT2, GFRA1, CALM2 |
| HFH1 | 29 | 53.70 | 0.02 | USP6, C6, PABPC4, EPHB2, APP, HIF1AN, CD44, BAG3, SERPINE1, SMAD9, MAN1A2, RYK, CREB1, COL15A1, SMAD3, ELAVL1, CFTR, FRG1B, HMGA1, STAT3, ANXA2, NPC1, HIF1A, EP300, VCP, INVS, NEUROD1, GFRA1, CALM2 |
| STAT3 | 30 | 55.56 | 0.02 | USP6, UBE3A, SART3, ITSN1, EPHB2, APP, CD44, SAA2, SAA1, BAG3, SERPINE1, VPS4A, LBP, SMAD9, IL8, CREB1, COL15A1, SMAD3, CFTR, HMGA1, STAT3, ANXA2, SDC1, HIF1A, EP300, INVS, DGAT2, NEUROD1, GFRA1, CALM2 |
| STAT | 24 | 44.44 | 0.02 | USP6, CXCL5, UBE3A, RYK, CREB1, C6, RELA, COL15A1, SMAD3, FRG1B, ITSN1, STAT3, CXCL10, EPHB2, ORM1, SDC1, SP1, CD44, VCP, BAG3, VPS4A, GFRA1, NEUROD1, FN1 |
| TBP | 24 | 44.44 | 0.02 | USP6, SMAD9, MAN1A2, UBE3A, CREB1, PABPC4, SMAD3, ARF6, FRG1B, ITSN1, STAT3, ANXA2, EPHB2, APP, EP300, HIF1A, CD44, INVS, HIF1AN, VPS4A, GFRA1, NEUROD1, IGFBP1, CALM2 |
| FAC1 | 30 | 55.56 | 0.02 | UBE3A, ARF6, SART3, ITSN1, SRC, EPHB2, APP, HIF1AN, CD44, SERPINE1, FN1, SMAD9, MAN1A2, CREB1, RELA, COL15A1, SMAD3, CFTR, STAT3, ANXA2, SDC1, NPC1, HIF1A, EP300, SP1, INVS, DGAT2, NEUROD1, GFRA1, CALM2 |
| FOXO4 | 33 | 61.11 | 0.02 | USP6, UBE3A, PABPC4, SART3, ITSN1, SRC, EPHB2, APP, CD44, HIF1AN, BAG3, LBP, FN1, MAN1A2, RYK, CREB1, COL15A1, SMAD3, ELAVL1, CFTR, STAT3, ANXA2, SDC1, HIF1A, EP300, SP1, VCP, INVS, DGAT2, NEUROD1, GFRA1, IGFBP1, CALM2 |
| HSF2 | 27 | 50.00 | 0.02 | UBE3A, C6, ARF6, SART3, EPHB2, CXCL10, APP, BAG1, CD44, SAA1, SERPINE1, VPS4A, LBP, FN1, MAN1A2, RELA, COL15A1, SMAD3, CFTR, STAT3, ANXA2, SDC1, VCP, INVS, DGAT2, NEUROD1, GFRA1 |
| HEN1 | 35 | 64.81 | 0.02 | USP6, CXCL5, UBE3A, PABPC4, CXCR2, SART3, ITSN1, SRC, EPHB2, CD44, BAG1, SAA1, BAG3, SERPINE1, VPS4A, LBP, CEBPB, SMAD9, CREB1, RELA, COL15A1, SMAD3, HMGA1, STAT3, ANXA2, SDC1, HIF1A, EP300, SP1, VCP, INVS, DGAT2, GFRA1, IGFBP1, CALM2 |
| HNF1 | 33 | 61.11 | 0.03 | USP6, UBE3A, C6, PABPC4, ARF6, SART3, ITSN1, SRC, EPHB2, APP, CD44, HIF1AN, FN1, MAN1A2, RYK, CREB1, SMAD3, ELAVL1, FRG1B, CFTR, HMGA1, STAT3, ANXA2, SDC1, NPC1, HIF1A, SP1, INVS, DGAT2, NEUROD1, GFRA1, IGFBP1, CALM2 |
| LHX3 | 24 | 44.44 | 0.03 | USP6, RYK, CREB1, C6, COL15A1, SMAD3, ARF6, ITSN1, HMGA1, STAT3, CXCL10, EPHB2, ANXA2, APP, HIF1A, EP300, CD44, BAG1, INVS, HIF1AN, NEUROD1, GFRA1, LBP, FN1 |
| IRF1 | 18 | 33.33 | 0.04 | CREB1, C6, RELA, PABPC4, COL15A1, SMAD3, ELAVL1, FRG1B, ITSN1, HMGA1, CXCL10, SDC1, APP, INVS, CD44, SERPINE1, GFRA1, NEUROD1 |
| E4BP4 | 27 | 50.00 | 0.04 | USP6, CXCL5, UBE3A, C6, ITSN1, SART3, APP, CD44, BAG3, LBP, FN1, MAN1A2, CREB1, COL15A1, SMAD3, ELAVL1, FRG1B, HMGA1, STAT3, ANXA2, HIF1A, EP300, INVS, DGAT2, GFRA1, NEUROD1, CALM2 |
| HOXA3 | 26 | 48.15 | 0.04 | USP6, UBE3A, C6, ITSN1, SART3, SRC, EPHB2, APP, HIF1AN, SAA2, CD44, SAA1, BAG3, SERPINE1, VPS4A, FN1, MAN1A2, RYK, SMAD3, STAT3, EP300, VCP, INVS, SP1, GFRA1, CALM2 |
| FOXO1 | 26 | 48.15 | 0.04 | USP6, UBE3A, C6, ITSN1, SRC, EPHB2, APP, BAG1, SAA2, CD44, BAG3, SMAD9, MAN1A2, RYK, CREB1, COL15A1, SMAD3, ELAVL1, CFTR, SDC1, EP300, INVS, SP1, GFRA1, NEUROD1, CALM2 |
| CP2 | 25 | 46.30 | 0.04 | MAN1A2, RYK, UBE3A, RELA, C6, SMAD3, ELAVL1, ITSN1, HMGA1, STAT3, SRC, ANXA2, EPHB2, NPC1, APP, SDC1, EP300, HIF1A, HIF1AN, DGAT2, INVS, BAG3, GFRA1, CALM2, FN1 |
| S8 | 28 | 51.85 | 0.04 | CXCL5, UBE3A, C6, ITSN1, EPHB2, APP, BAG1, CD44, BAG3, FN1, MAN1A2, RYK, COL15A1, SMAD3, ELAVL1, HMGA1, STAT3, ANXA2, SDC1, HIF1A, EP300, SP1, VCP, INVS, DGAT2, GFRA1, NEUROD1, CALM2 |
| NFKAPPAB | 23 | 42.59 | 0.04 | CXCL5, IL8, RYK, CREB1, RELA, PABPC4, COL15A1, CFTR, SART3, SRC, STAT3, CXCL10, EPHB2, ANXA2, HIF1A, SP1, CD44, DGAT2, BAG1, HIF1AN, NEUROD1, GFRA1, LBP |
| NFKB | 30 | 55.56 | 0.04 | CXCL5, C6, PABPC4, SART3, ITSN1, SRC, EPHB2, CXCL10, APP, CD44, HIF1AN, SERPINE1, LBP, FN1, SMAD9, IL8, RYK, CREB1, COL15A1, SMAD3, CFTR, HMGA1, STAT3, ANXA2, SP1, VCP, DGAT2, NEUROD1, GFRA1, CALM2 |
| MYCMAX | 36 | 66.67 | 0.04 | USP6, PABPC4, ARF6, SART3, ITSN1, EPHB2, APOA1, CD44, BAG1, BAG3, SERPINE1, VPS4A, FN1, SMAD9, CEBPB, MAN1A2, CREB1, RELA, COL15A1, SMAD3, ELAVL1, FRG1B, CFTR, HMGA1, STAT3, ANXA2, SDC1, NPC1, HIF1A, EP300, VCP, INVS, DGAT2, NEUROD1, GFRA1, CALM2 |
| ISRE | 27 | 50.00 | 0.04 | CXCL5, UBE3A, C6, ARF6, ITSN1, SART3, CXCL10, APP, BAG1, SERPINE1, LBP, FN1, RYK, CREB1, SMAD3, CFTR, FRG1B, HMGA1, STAT3, NPC1, HIF1A, EP300, INVS, SP1, GFRA1, NEUROD1, CALM2 |
| SPZ1 | 21 | 38.89 | 0.05 | UBE3A, RYK, CREB1, RELA, SMAD3, CFTR, SART3, HMGA1, SRC, EPHB2, ORM1, SDC1, EP300, SP1, CD44, HIF1AN, VCP, BAG3, SERPINE1, GFRA1, CALM2 |
| OCT | 28 | 51.85 | 0.05 | USP6, UBE3A, C6, PABPC4, SART3, ITSN1, SRC, EPHB2, APP, BAG1, HIF1AN, CD44, BAG3, VPS4A, FN1, MAN1A2, RYK, CREB1, SMAD3, CFTR, HMGA1, ANXA2, HIF1A, EP300, VCP, INVS, NEUROD1, GFRA1 |
| TAL1ALPHAE47 | 15 | 27.78 | 0.05 | UBE3A, RYK, SMAD3, ELAVL1, STAT3, ANXA2, EPHB2, APP, SDC1, EP300, HIF1AN, CD44, SP1, GFRA1, CALM2 |
| GFI1 | 27 | 50.00 | 0.05 | USP6, SMAD9, MAN1A2, RYK, CREB1, PABPC4, COL15A1, SMAD3, CFTR, ITSN1, HMGA1, CXCL10, ANXA2, EPHB2, NPC1, APP, EP300, HIF1A, CD44, HIF1AN, INVS, VCP, GFRA1, NEUROD1, IGFBP1, CALM2, FN1 |

* The number of submitted genes that can be regulated by each TF over the total number of genes that can be regulated by each TF

**Supplementary Table 4. List of BA patient samples used in each data source**

| **Patient #** | **GWAS-family trio-TDT** | **Target sequencing** | **Whole exome sequencing** | **RNAseq** |
| --- | --- | --- | --- | --- |
| 1 | Y | Y | Y |  |
| 2 | Y |  |  |  |
| 3 | Y | Y | Y |  |
| 4 | Y | Y | Y |  |
| 5 | Y | Y | Y |  |
| 6 | Y | Y | R |  |
| 7 | Y | Y | R |  |
| 8 | Y |  |  |  |
| 9 | Y |  |  |  |
| 10 | Y | Y | Y |  |
| 11 | Y | Y | Y |  |
| 12 | Y | Y | Y |  |
| 13 | Y | Y | Y |  |
| 14 | Y |  | R |  |
| 15 | Y |  |  |  |
| 16 | Y |  |  |  |
| 17 | Y | Y | Y |  |
| 18 | Y | Y | Y |  |
| 19 | Y | Y | Y |  |
| 20 | Y | Y | R |  |
| 21 | Y |  |  |  |
| 22 | Y | Y | Y |  |
| 23 | Y |  |  |  |
| 24 | Y | Y | Y |  |
| 25 | Y | Y | Y | Y |
| 26 | Y | Y | Y | Y |
| 27 | Y | Y | Y | Y |
| 28 | Y | Y | Y | Y |
| 29 | Y | Y | R |  |
| 30 | Y |  |  |  |
| 31 | Y | Y | Y |  |
| 32 | Y | Y | Y |  |
| 33 | Y | Y | Y |  |
| 34 | Y |  |  |  |
| 35 | Y | Y | Y |  |
| 36 | Y |  | Y |  |
| 37 |  | Y | Y |  |
| 38 |  |  | Y |  |
| 39 |  | Y | Y |  |
| 40 |  | Y | Y |  |
| 41 |  | Y | Y |  |
| 42 |  | Y | Y |  |
| 43 |  |  | Y |  |
| 44 |  | Y | Y |  |
| 45 |  | Y | Y |  |
| 46 |  | Y | Y |  |
| 47 |  | Y | Y |  |
| 48 |  | Y | Y |  |
| 49 |  |  | Y |  |
| 50 |  | Y | Y |  |
| 51 |  | Y | Y |  |
| 52 |  | Y | Y |  |
| 53 |  | Y | Y | Y |
| 54 |  |  | Y |  |
| 55 |  | Y | Y |  |
| 56 |  |  | Y |  |
| 57 |  |  | Y |  |
| 58 |  |  | Y |  |
| 59 |  |  | Y |  |
| 60 |  | Y | Y |  |
| 61 |  |  | Y |  |
| 62 |  | Y | Y |  |
| 63 |  | Y | Y | Y |
| Total | 36 | 43 | 49 | 6 |

*Y=Yes for patient sample used, R=removed due to low sequencing depth

**Supplementary Table 5. Comparison of the RNAseq results to the published literature**

The RNAseq results were compared to the published data from Bessho et. al from *Hetatology* investigating the role of IL8 in BA. 172 genes were common from our list of differentially regulated genes (p<0.05) and their list of differentially regulated genes with more than at least 2 fold changes. Among these, 10 genes were also part of the integrative analysis results.

| **Gene** | **P-value** |
| --- | --- |
| 7A5 | 0.040116 |
| ACACB | 0.000514 |
| ACSS2 | 0.008745 |
| ADH4 | 0.002759 |
| ADRA1A | 0.020002 |
| AGPAT9 | 0.036992 |
| AGXT2 | 0.004327 |
| AKR1B10 | 1.32E-09 |
| AKR1C4 | 0.032684 |
| AKR1D1 | 3.53E-05 |
| ANKRD1 | 5.13E-06 |
| ANTXR1 | 0.002465 |
| ANXA1* | 0.032318 |
| ANXA13 | 0.019705 |
| ANXA2 | 0.037905 |
| AOX1 | 0.010286 |
| ASPN | 0.02715 |
| AVPR1A | 1.1E-05 |
| B3GNT5 | 0.002055 |
| BBOX1 | 9.33E-05 |
| BICC1 | 0.004451 |
| C13orf15 | 0.014103 |
| C4BPB | 0.029266 |
| C6* | 0.0134 |
| C9 | 0.015491 |
| CA12 | 0.000373 |
| CAPG | 5.37E-05 |
| CCDC80 | 0.001561 |
| CCL18 | 0.001193 |
| CCL20 | 0.000339 |
| CD24 | 0.038328 |
| CDH6 | 0.002545 |
| CES1 | 0.025111 |
| CFHR4 | 0.013927 |
| CFHR5 | 0.014634 |
| CFTR* | 0.000194 |
| CLIC6 | 0.000154 |
| CNDP1 | 2.03E-06 |
| COL6A3 | 0.036214 |
| CRP | 0.000511 |
| CTGF | 0.014544 |
| CXCL10 | 1.16E-05 |
| CXCL6 | 0.00013 |
| CXCR4 | 0.001084 |
| CYP1A2 | 6.1E-06 |
| DCDC2 | 0.000177 |
| DGAT2* | 0.000995 |
| DHODH | 0.000454 |
| DHRS13 | 0.022466 |
| DTNA | 0.005719 |
| DTX4 | 0.009965 |
| EFEMP1 | 0.002766 |
| ELOVL6 | 0.021947 |
| ELOVL7 | 0.016085 |
| EMP1 | 0.03529 |
| FAP | 6.02E-05 |
| FAT | 0.004051 |
| FCN3 | 0.039643 |
| FDPS | 0.034226 |
| GBA3 | 0.014779 |
| GDF15 | 0.000638 |
| GEM | 0.000636 |
| GFRA1* | 0.016235 |
| GLYATL1 | 0.017448 |
| GNAO1 | 0.000608 |
| GNMT | 0.014884 |
| GPNMB | 0.000266 |
| GPR125 | 0.034772 |
| GPRIN3 | 0.010063 |
| GPX2 | 0.033624 |
| HAMP | 0.006128 |
| HAO2 | 4.29E-07 |
| HBB | 2.32E-09 |
| HLA-DMB | 0.027684 |
| HMGCS1 | 0.009984 |
| HP | 6.15E-05 |
| HPR | 0.015815 |
| HPX | 0.007831 |
| HSD17B6 | 0.016424 |
| IDH2 | 0.012961 |
| IGF1 | 0.032103 |
| IGF2 | 0.010096 |
| IGSF9 | 0.000474 |
| IL32 | 0.009944 |
| IL8 | 3.15E-05 |
| ITGA2 | 0.015142 |
| ITGAX | 0.014785 |
| ITGB8 | 0.022791 |
| KCNK5 | 0.023677 |
| KIAA0152 | 0.005462 |
| KIAA0746 | 0.019604 |
| KRT19 | 6.24E-06 |
| KRT23 | 1.71E-07 |
| KRT7 | 1.18E-06 |
| LAMC2 | 1.13E-07 |
| LBP* | 0.029103 |
| LOC388503 | 0.049826 |
| LRG1 | 0.002417 |
| LRIG1 | 0.032872 |
| LSS | 0.007717 |
| LUM | 0.000113 |
| MAT1A | 0.032454 |
| MFAP3L | 0.013276 |
| MGP* | 0.000122 |
| MMP19 | 0.038732 |
| MMP2 | 0.010307 |
| MMP7 | 3.24E-09 |
| MOCOS | 0.029745 |
| MOGAT2 | 0.020534 |
| MOXD1 | 4.39E-05 |
| MT1M | 0.038111 |
| OLR1 | 0.000295 |
| ORM1 | 0.000482 |
| ORM2 | 0.005258 |
| PCOLCE2 | 0.010204 |
| PDGFD | 0.038181 |
| PFKFB3 | 0.006245 |
| PHGDH | 0.039321 |
| PKHD1 | 0.025658 |
| PLA2G7 | 0.010105 |
| PLAT | 0.006135 |
| PMP22 | 0.029402 |
| PRG4 | 0.000519 |
| PROM1 | 0.010774 |
| PSAT1 | 0.004074 |
| PZP | 5.12E-06 |
| RAB11FIP1 | 0.020552 |
| RDH16 | 0.002686 |
| RGS1 | 0.000263 |
| RGS4 | 2.89E-05 |
| RND2 | 0.000783 |
| SAA1 | 3.68E-08 |
| SC5DL | 0.038108 |
| SCRN1 | 0.023977 |
| SCTR | 0.002707 |
| SELENBP1 | 0.037947 |
| SERPINA10 | 0.01026 |
| SERPINA3 | 0.000992 |
| SERPINE2 | 0.011484 |
| SGIP1 | 0.000446 |
| SLC12A2 | 0.004768 |
| SLC17A2 | 0.001129 |
| SLC22A1 | 0.03518 |
| SLC28A1* | 0.013273 |
| SLC2A10 | 0.014276 |
| SLC2A3 | 0.003159 |
| SLC44A3 | 0.005927 |
| SLCO4C1* | 0.000442 |
| SPINK1 | 0.000237 |
| SPP1 | 0.00011 |
| SQLE | 0.014816 |
| STARD5 | 0.011132 |
| STEAP3 | 0.013069 |
| STK39 | 0.015746 |
| STMN2 | 0.008711 |
| SULF1 | 0.013184 |
| SULT1C4 | 0.040311 |
| TACSTD1 | 0.000751 |
| TACSTD2 | 2.2E-06 |
| THBS2 | 0.002857 |
| THRSP | 0.002973 |
| THY1 | 0.00207 |
| TM4SF1 | 0.009111 |
| TMEM156 | 0.014849 |
| TMEM97 | 0.046299 |
| TMSB10 | 0.039524 |
| TNFAIP3 | 0.000584 |
| TTPA | 0.024716 |
| VCAN | 0.002936 |
| VIM* | 0.016495 |
| VTCN1 | 7.57E-05 |
| WDR72 | 0.00033 |

* Genes that also show up in the set-based test in PLINK for the integrative result

**Supplementary Table 6. List of differentially regulated genes from the RNAseq data**

| **Gene** | **Fold Change** | **P-value** | **Adjusted P-value** |
| --- | --- | --- | --- |
| AKR1B10 | 265.4259 | 1.32E-09 | 1.17E-05 |
| HBB | 116.3477 | 2.32E-09 | 1.17E-05 |
| MMP7 | 177.2804 | 3.24E-09 | 1.17E-05 |
| HBA2 | 123.4131 | 7.89E-09 | 2.14E-05 |
| HBA1 | 193.1111 | 1.01E-08 | 2.20E-05 |
| SAA1 | 0.015433 | 3.68E-08 | 6.47E-05 |
| SAA2 | 0.015718 | 4.18E-08 | 6.47E-05 |
| KRT17 | 361.7778 | 8.23E-08 | 0.000112 |
| LAMC2 | 205.3333 | 1.13E-07 | 0.000137 |
| KRT23 | 101.037 | 1.71E-07 | 0.000186 |
| HAO2 | 0.019372 | 4.29E-07 | 0.000424 |
| SFRP4 | Inf | 5.47E-07 | 0.000494 |
| KRT7 | 41.53292 | 1.18E-06 | 0.000987 |
| MUC13 | 65.06878 | 1.72E-06 | 0.001335 |
| CNDP1 | 0.009155 | 2.03E-06 | 0.001468 |
| TACSTD2 | 58.87037 | 2.20E-06 | 0.001488 |
| CLDN4 | 58.43386 | 3.36E-06 | 0.002142 |
| STC1 | 38.2963 | 3.72E-06 | 0.002244 |
| TESC | 89.35802 | 4.55E-06 | 0.00242 |
| CGA | Inf | 4.81E-06 | 0.00242 |
| FGF23 | 74.14815 | 4.86E-06 | 0.00242 |
| PZP | 0.029777 | 5.12E-06 | 0.00242 |
| ANKRD1 | 195.5556 | 5.13E-06 | 0.00242 |
| CYP1A2 | 0.024493 | 6.10E-06 | 0.002603 |
| CHIT1 | Inf | 6.15E-06 | 0.002603 |
| KRT19 | 34.1868 | 6.24E-06 | 0.002603 |
| SPINT1 | 52.14815 | 6.77E-06 | 0.002718 |
| GREM1 | 108.3704 | 7.65E-06 | 0.002963 |
| AVPR1A | 0.008954 | 1.10E-05 | 0.004108 |
| CXCL10 | 39.11111 | 1.16E-05 | 0.00419 |
| PRSS22 | Inf | 1.78E-05 | 0.006214 |
| RGS4 | 33.48148 | 2.89E-05 | 0.009799 |
| IL8 | 21.21281 | 3.15E-05 | 0.010198 |
| COMP | Inf | 3.20E-05 | 0.010198 |
| AKR1D1 | 0.035837 | 3.53E-05 | 0.010939 |
| GSTM1 | 0.030556 | 3.79E-05 | 0.011406 |
| LTBP2 | 29.77208 | 3.90E-05 | 0.011445 |
| MOXD1 | 51.94444 | 4.39E-05 | 0.012532 |
| CAPG | 22.36214 | 5.37E-05 | 0.014948 |
| TREM2 | 78.62963 | 5.68E-05 | 0.015393 |
| FAP | Inf | 6.02E-05 | 0.015879 |
| HP | 0.063091 | 6.15E-05 | 0.015879 |
| CHRNA4 | 0.022142 | 7.49E-05 | 0.018598 |
| VTCN1 | 74.96296 | 7.57E-05 | 0.018598 |
| STC2 | 47.25926 | 7.72E-05 | 0.018598 |
| CCL4 | 30.32922 | 8.43E-05 | 0.019874 |
| DDX3Y | 0.019874 | 8.74E-05 | 0.020162 |
| BBOX1 | 0.007341 | 9.33E-05 | 0.021085 |
| SPP1 | 15.17662 | 0.00011 | 0.024409 |
| LUM | 16.43419 | 0.000113 | 0.024409 |
| PDZK1IP1 | 52.69136 | 0.000118 | 0.025032 |
| MGP | 15.14023 | 0.000122 | 0.025536 |
| PMEPA1 | 19.90982 | 0.000128 | 0.026126 |
| CXCL6 | 26.80741 | 0.00013 | 0.026126 |
| UBD | 14.65519 | 0.000134 | 0.026222 |
| DKK1 | 115.7037 | 0.000135 | 0.026222 |
| HS3ST2 | Inf | 0.000147 | 0.027918 |
| CLIC6 | 36.82963 | 0.000154 | 0.028836 |
| CXCL5 | 111.6296 | 0.000167 | 0.030673 |
| USH2A | 0.008148 | 0.000174 | 0.031433 |
| DCDC2 | 23.49383 | 0.000177 | 0.031433 |
| CFTR | 22.37607 | 0.000194 | 0.033924 |
| ITGBL1 | 40.12963 | 0.000199 | 0.03429 |
| FGF19 | Inf | 0.000225 | 0.038143 |
| SPINK1 | 0.075957 | 0.000237 | 0.039079 |
| HBG2 | Inf | 0.000238 | 0.039079 |
| S100A6 | 13.0963 | 0.000262 | 0.040567 |
| CLDN7 | 16.05974 | 0.000263 | 0.040567 |
| RGS1 | 14.81152 | 0.000263 | 0.040567 |
| DBNDD1 | 24.71605 | 0.000265 | 0.040567 |
| GPNMB | 12.75607 | 0.000266 | 0.040567 |
| ADCY1 | 0.050007 | 0.000282 | 0.042539 |
| OLR1 | 101.037 | 0.000295 | 0.043801 |
| WDR72 | 0.052569 | 0.00033 | 0.048391 |
| CCL20 | 14.85291 | 0.000339 | 0.049094 |
| IGFALS | 0.07047 | 0.000362 | 0.051684 |
| RAMP1 | 12.85342 | 0.00037 | 0.051918 |
| CA12 | 31.45185 | 0.000373 | 0.051918 |
| SLCO4C1 | 0.009586 | 0.000442 | 0.060488 |
| SGIP1 | Inf | 0.000446 | 0.060488 |
| DHODH | 0.084702 | 0.000454 | 0.060776 |
| IGSF9 | 0.044044 | 0.000474 | 0.062739 |
| ORM1 | 0.095941 | 0.000482 | 0.062936 |
| CRP | 0.097128 | 0.000511 | 0.065509 |
| ACACB | 0.090268 | 0.000514 | 0.065509 |
| PRG4 | 0.090933 | 0.000519 | 0.065509 |
| TMEM132A | 22.71296 | 0.000566 | 0.070567 |
| TNFAIP3 | 11.20173 | 0.000584 | 0.071973 |
| GNAO1 | 0.061264 | 0.000608 | 0.072899 |
| SRD5A2 | 0.063342 | 0.00061 | 0.072899 |
| RNASE1 | 11.40741 | 0.000612 | 0.072899 |
| GEM | 12.80722 | 0.000636 | 0.074394 |
| GDF15 | 10.53987 | 0.000638 | 0.074394 |
| C13orf33 | 28.02963 | 0.000694 | 0.080135 |
| PADI1 | 0 | 0.000716 | 0.081727 |
| TACSTD1 | 18.59259 | 0.000751 | 0.084817 |
| RND2 | 0.059259 | 0.000783 | 0.087602 |
| SYT13 | Inf | 0.000879 | 0.097239 |
| SERPINA3 | 0.111911 | 0.000992 | 0.107901 |
| DGAT2 | 0.104755 | 0.000995 | 0.107901 |
| ITIH5 | 19.10288 | 0.001039 | 0.11132 |
| CTHRC1 | 79.85185 | 0.001047 | 0.11132 |
| CXCR4 | 10.70899 | 0.001084 | 0.114149 |
| SLC17A2 | 0.091606 | 0.001129 | 0.117677 |
| PTHLH | Inf | 0.001139 | 0.117677 |
| CCL18 | 25.25926 | 0.001193 | 0.121001 |
| COL7A1 | 0.069941 | 0.001194 | 0.121001 |
| F2RL3 | 28.72222 | 0.001214 | 0.121977 |
| HSPA1B | 9.506173 | 0.001269 | 0.126248 |
| FSTL3 | 9.833587 | 0.001336 | 0.131727 |
| GPRC5A | 17.27407 | 0.001352 | 0.132071 |
| MMP1 | Inf | 0.001391 | 0.134727 |
| NCAM1 | 17.92593 | 0.001432 | 0.137412 |
| ITGA3 | 11.97151 | 0.001462 | 0.13909 |
| SELE | 12.48148 | 0.001522 | 0.14352 |
| CCDC80 | 10.29147 | 0.001561 | 0.145939 |
| SEZ6L2 | 12.57143 | 0.001579 | 0.146356 |
| C3orf25 | 21.45679 | 0.001626 | 0.149457 |
| BACE2 | 10.76035 | 0.001736 | 0.158205 |
| PID1 | 0.107843 | 0.002012 | 0.181906 |
| B3GNT5 | 18.85714 | 0.002055 | 0.184006 |
| THY1 | 9.34127 | 0.00207 | 0.184006 |
| OXTR | Inf | 0.002103 | 0.184501 |
| PAPLN | 16.5679 | 0.002109 | 0.184501 |
| EEF1A2 | 40.33333 | 0.00223 | 0.193187 |
| GSTA2 | 0.115383 | 0.002244 | 0.193187 |
| PDGFA | 11.30113 | 0.002292 | 0.195796 |
| LRG1 | 0.135084 | 0.002417 | 0.204101 |
| GHR | 0.121355 | 0.002451 | 0.204101 |
| COL10A1 | 39.51852 | 0.002461 | 0.204101 |
| ANTXR1 | 9.053498 | 0.002465 | 0.204101 |
| FADS1 | 0.124599 | 0.002538 | 0.205976 |
| RXRA | 0.133304 | 0.002541 | 0.205976 |
| CDH6 | 18.04233 | 0.002545 | 0.205976 |
| HNT | 66 | 0.00268 | 0.214259 |
| RDH16 | 0.135031 | 0.002686 | 0.214259 |
| SCTR | 17.80952 | 0.002707 | 0.21436 |
| ADH4 | 0.137779 | 0.002759 | 0.215859 |
| EFEMP1 | 9.10467 | 0.002766 | 0.215859 |
| THBS2 | 8.062378 | 0.002857 | 0.22137 |
| FOXP3 | 24.03704 | 0.002919 | 0.224258 |
| VCAN | 8.555556 | 0.002936 | 0.224258 |
| THRSP | 0.112112 | 0.002973 | 0.225477 |
| DEFB1 | 7.606878 | 0.003109 | 0.234205 |
| SLC2A3 | 7.9702 | 0.003159 | 0.236329 |
| SLC30A2 | Inf | 0.003243 | 0.240919 |
| LGALS3 | 7.94709 | 0.003444 | 0.254124 |
| CYP2E1 | 0.147629 | 0.003489 | 0.25573 |
| ISLR | 9.076132 | 0.003591 | 0.261411 |
| NDST1 | 0.143521 | 0.003819 | 0.276137 |
| IHPK3 | 0.097628 | 0.003871 | 0.278086 |
| KCNE4 | 15.27778 | 0.004046 | 0.286448 |
| FAT | 7.071605 | 0.004051 | 0.286448 |
| PSAT1 | 0.129215 | 0.004074 | 0.286448 |
| B3GNT3 | 14.39506 | 0.004093 | 0.286448 |
| IL6 | 9.68 | 0.004172 | 0.288367 |
| MME | 0.067901 | 0.004174 | 0.288367 |
| DDR1 | 8.698699 | 0.004223 | 0.289094 |
| EGLN3 | 16.17989 | 0.004238 | 0.289094 |
| AGXT2 | 0.138122 | 0.004327 | 0.293042 |
| PCSK1N | 12.96296 | 0.00435 | 0.293042 |
| BICC1 | 11.35309 | 0.004451 | 0.298008 |
| DPEP1 | 34.62963 | 0.004548 | 0.30265 |
| CTSK | 9.671498 | 0.004694 | 0.310454 |
| FGG | 0.1588 | 0.004736 | 0.311361 |
| SLC12A2 | 7.769841 | 0.004768 | 0.311566 |
| PDLIM4 | 18.9037 | 0.004812 | 0.312559 |
| BDKRB2 | 13.76132 | 0.005021 | 0.323321 |
| RRAD | 12.08642 | 0.005037 | 0.323321 |
| ORM2 | 0.162039 | 0.005258 | 0.333983 |
| SDCBP2 | 9.302469 | 0.005265 | 0.333983 |
| KIAA0152 | 0.15876 | 0.005462 | 0.344446 |
| FGB | 0.164735 | 0.005538 | 0.347201 |
| DTNA | 11.74691 | 0.005719 | 0.355502 |
| INHBA | 8.812071 | 0.005735 | 0.355502 |
| CCL3 | 7.981007 | 0.005852 | 0.360693 |
| ADH6 | 0.159669 | 0.005925 | 0.361179 |
| SLC44A3 | 15.01587 | 0.005927 | 0.361179 |
| HAMP | 0.167633 | 0.006128 | 0.369666 |
| PLAT | 9.692008 | 0.006135 | 0.369666 |
| ARL4D | 0.126627 | 0.006184 | 0.369666 |
| PHLDA3 | 24.44444 | 0.006203 | 0.369666 |
| PFKFB3 | 6.407865 | 0.006245 | 0.370176 |
| PAQR5 | 32.18519 | 0.006284 | 0.370436 |
| GATA4 | 0.148025 | 0.006362 | 0.3729 |
| COL16A1 | 9.777778 | 0.006411 | 0.3729 |
| KRT81 | Inf | 0.006463 | 0.3729 |
| FA2H | Inf | 0.006463 | 0.3729 |
| MUC20 | 9.73251 | 0.006542 | 0.375457 |
| INHBC | 0.147141 | 0.006785 | 0.387365 |
| FLJ45139 | 0.02859 | 0.006926 | 0.391405 |
| IFI6 | 6.140351 | 0.006943 | 0.391405 |
| COL11A1 | Inf | 0.007 | 0.391405 |
| WNT10A | Inf | 0.007 | 0.391405 |
| LRRC50 | 52.96296 | 0.007159 | 0.39815 |
| ISG15 | 6.864198 | 0.00723 | 0.39815 |
| HES4 | 8.888889 | 0.007231 | 0.39815 |
| SYT7 | 0.164011 | 0.007428 | 0.406699 |
| ALAS2 | 17.11111 | 0.007461 | 0.406699 |
| LSS | 0.165402 | 0.007717 | 0.418533 |
| HPX | 0.178611 | 0.007831 | 0.422596 |
| PPAP2C | 10.52991 | 0.00796 | 0.426975 |
| TNFRSF12A | 6.277092 | 0.007991 | 0.426975 |
| BATF | 13.73545 | 0.008711 | 0.459299 |
| STMN2 | 50.51852 | 0.008711 | 0.459299 |
| ACSS2 | 0.172115 | 0.008745 | 0.459299 |
| EPS8L1 | 22.54321 | 0.008765 | 0.459299 |
| HSPB8 | 8.613757 | 0.008817 | 0.459804 |
| TM4SF1 | 5.639797 | 0.009111 | 0.4717 |
| HTRA3 | 9.89418 | 0.009132 | 0.4717 |
| CFHR3 | 0.172303 | 0.00919 | 0.472315 |
| SPRY1 | 6.753561 | 0.009231 | 0.472315 |
| FADS2 | 0.177867 | 0.009442 | 0.479815 |
| CACNA1H | 0.146719 | 0.009668 | 0.479815 |
| PFKP | 7.71164 | 0.009716 | 0.479815 |
| PTGDS | 6.773148 | 0.009735 | 0.479815 |
| CRYAB | 11.24444 | 0.009745 | 0.479815 |
| FABP4 | 11.76955 | 0.00988 | 0.479815 |
| IL32 | 5.387507 | 0.009944 | 0.479815 |
| CCDC69 | 0.164191 | 0.009947 | 0.479815 |
| DTX4 | 0.158804 | 0.009965 | 0.479815 |
| HMGCS1 | 0.182478 | 0.009984 | 0.479815 |
| ARRDC2 | 6.218324 | 0.010002 | 0.479815 |
| GPRIN3 | 13.26984 | 0.010063 | 0.479815 |
| IGF2 | 0.189144 | 0.010096 | 0.479815 |
| PLA2G7 | 7.803419 | 0.010105 | 0.479815 |
| PLAUR | 6.533333 | 0.010144 | 0.479815 |
| GFPT2 | 10.66667 | 0.010166 | 0.479815 |
| PCOLCE2 | 0.087019 | 0.010204 | 0.479815 |
| SERPINA10 | 0.186921 | 0.01026 | 0.479815 |
| S100A11 | 5.737189 | 0.010284 | 0.479815 |
| AOX1 | 0.189407 | 0.010286 | 0.479815 |
| MMP2 | 5.956296 | 0.010307 | 0.479815 |
| C10orf132 | Inf | 0.010544 | 0.488772 |
| LPL | 48.07407 | 0.010642 | 0.491215 |
| PROM1 | 8.491228 | 0.010774 | 0.495213 |
| HSPA1A | 5.626348 | 0.010846 | 0.495391 |
| CD52 | 7.077249 | 0.01087 | 0.495391 |
| STARD5 | 0.135802 | 0.011132 | 0.505225 |
| ASPHD1 | 21.18519 | 0.011312 | 0.511234 |
| SERPINE2 | 7.962963 | 0.011484 | 0.516862 |
| SPINT2 | 6.57284 | 0.011638 | 0.519946 |
| IGFBP7 | 5.15018 | 0.011648 | 0.519946 |
| VSTM2L | 17.51852 | 0.011712 | 0.520668 |
| PRDM1 | 15.31852 | 0.011834 | 0.523918 |
| DKK3 | 6.330484 | 0.012055 | 0.530102 |
| AR | 0.126857 | 0.012071 | 0.530102 |
| SEMA3C | 46.44444 | 0.01219 | 0.533164 |
| SOX9 | 7.604938 | 0.012398 | 0.540103 |
| LOC55908 | 5.55677 | 0.012534 | 0.543845 |
| MBNL3 | 0.137232 | 0.012874 | 0.553491 |
| JMJD5 | 0.166864 | 0.012916 | 0.553491 |
| CXCL1 | 8.854321 | 0.012918 | 0.553491 |
| IDH2 | 0.197401 | 0.012961 | 0.553491 |
| STEAP3 | 0.19625 | 0.013069 | 0.553852 |
| KLHL6 | 11.61111 | 0.013179 | 0.553852 |
| SULF1 | 7.398519 | 0.013184 | 0.553852 |
| C15orf52 | 10 | 0.013235 | 0.553852 |
| SLC28A1 | 0.146813 | 0.013273 | 0.553852 |
| MFAP3L | 0.159056 | 0.013276 | 0.553852 |
| C6 | 0.20106 | 0.0134 | 0.556914 |
| MST150 | 9.276353 | 0.013465 | 0.557466 |
| CFHR4 | 0.129748 | 0.013927 | 0.572611 |
| PDPN | 44.81481 | 0.013989 | 0.572611 |
| AP1M2 | 44.81481 | 0.013989 | 0.572611 |
| HIPK2 | 0.191196 | 0.014042 | 0.572614 |
| C13orf15 | 6.686275 | 0.014103 | 0.572923 |
| SLC2A10 | 0.178987 | 0.014276 | 0.577817 |
| PTHR1 | 0.165093 | 0.014435 | 0.578063 |
| FGA | 0.207939 | 0.014447 | 0.578063 |
| CTGF | 5.152137 | 0.014544 | 0.578063 |
| CFHR5 | 0.183258 | 0.014634 | 0.578063 |
| HS3ST1 | 19.82716 | 0.0147 | 0.578063 |
| GBA3 | 0.147785 | 0.014779 | 0.578063 |
| ITGAX | 5.975309 | 0.014785 | 0.578063 |
| SQLE | 0.17505 | 0.014816 | 0.578063 |
| TMEM156 | 26.07407 | 0.014849 | 0.578063 |
| AQP1 | 5.28098 | 0.014871 | 0.578063 |
| GNMT | 0.193873 | 0.014884 | 0.578063 |
| HK2 | 9.703704 | 0.014922 | 0.578063 |
| DBN1 | 6.05291 | 0.015128 | 0.582445 |
| ITGA2 | 10.59259 | 0.015142 | 0.582445 |
| C9 | 0.209931 | 0.015491 | 0.593765 |
| STK39 | 11.87302 | 0.015746 | 0.601387 |
| HPR | 0.205311 | 0.015815 | 0.601915 |
| IGFBP5 | 4.929485 | 0.015956 | 0.605163 |
| ELOVL7 | 43.18519 | 0.016085 | 0.605815 |
| CA9 | 43.18519 | 0.016085 | 0.605815 |
| GFRA1 | 0.156266 | 0.016235 | 0.609354 |
| HSD17B6 | 0.209708 | 0.016424 | 0.612124 |
| VIM | 4.708216 | 0.016495 | 0.612124 |
| CPN1 | 0.203704 | 0.01651 | 0.612124 |
| JARID1D | 0.035427 | 0.016535 | 0.612124 |
| CNTFR | 0.047009 | 0.016609 | 0.612771 |
| CYP2C18 | 0.181302 | 0.016675 | 0.613118 |
| APOD | 14.01481 | 0.016801 | 0.613609 |
| SMOC2 | 14.01481 | 0.016801 | 0.613609 |
| SLC13A5 | 0.21356 | 0.016886 | 0.614647 |
| KNDC1 | 0.106996 | 0.017369 | 0.630087 |
| GLYATL1 | 0.204496 | 0.017448 | 0.630684 |
| SERINC5 | 0.158329 | 0.017501 | 0.630684 |
| SLCO1B3 | 0.183054 | 0.017944 | 0.644499 |
| S100P | 0.174805 | 0.018102 | 0.648018 |
| DUSP5 | 4.818342 | 0.018172 | 0.6484 |
| CXCR7 | 4.908174 | 0.01833 | 0.6519 |
| PRELP | 5.317189 | 0.01848 | 0.652018 |
| ACP5 | 4.820031 | 0.018493 | 0.652018 |
| DNAJC3 | 0.176403 | 0.018536 | 0.652018 |
| CLDN10 | 10.04938 | 0.018574 | 0.652018 |
| SNTB1 | 0.202863 | 0.018675 | 0.653459 |
| SCD5 | 10.59259 | 0.018865 | 0.657983 |
| PTGS2 | 6.111111 | 0.019017 | 0.661135 |
| ETV4 | 13.52593 | 0.019226 | 0.66465 |
| EPHB2 | 18.46914 | 0.01924 | 0.66465 |
| LSP1 | 5.229255 | 0.019579 | 0.672937 |
| KIAA0746 | 5.398148 | 0.019604 | 0.672937 |
| ANXA13 | 6.681481 | 0.019705 | 0.674268 |
| ABLIM2 | 40.74074 | 0.019905 | 0.674813 |
| ADM | 4.8318 | 0.019922 | 0.674813 |
| ADRA1A | 0.153377 | 0.020002 | 0.674813 |
| NEU4 | 0.17243 | 0.020029 | 0.674813 |
| CXCL9 | 7.022928 | 0.020065 | 0.674813 |
| DARC | 24.03704 | 0.020128 | 0.674813 |
| LIPG | 0.19464 | 0.020157 | 0.674813 |
| FMOD | 5.278583 | 0.020403 | 0.680963 |
| MOGAT2 | 0.124791 | 0.020534 | 0.681751 |
| RAB11FIP1 | 5.137308 | 0.020552 | 0.681751 |
| TMEM149 | 0.187457 | 0.02081 | 0.687684 |
| EIF1AY | 0.076389 | 0.020858 | 0.687684 |
| ATP6V0E2 | 0.182033 | 0.020934 | 0.688108 |
| CLCF1 | 8.273504 | 0.02103 | 0.689148 |
| CRYAA | 5.666667 | 0.021334 | 0.693382 |
| TXNIP | 4.50663 | 0.02135 | 0.693382 |
| BHLHB3 | 39.92593 | 0.021391 | 0.693382 |
| C20orf103 | 23.62963 | 0.021414 | 0.693382 |
| CLEC11A | 5.764815 | 0.021643 | 0.698681 |
| ELOVL6 | 0.198198 | 0.021947 | 0.70641 |
| ANXA3 | 9.125926 | 0.022237 | 0.713612 |
| GALNT2 | 0.226889 | 0.022387 | 0.714634 |
| TBC1D16 | 0.162037 | 0.022431 | 0.714634 |
| DHRS13 | 0.190811 | 0.022466 | 0.714634 |
| TTC9 | 6.703704 | 0.022613 | 0.717198 |
| TUBB3 | 11.67901 | 0.022789 | 0.718658 |
| ITGB8 | 23.22222 | 0.022791 | 0.718658 |
| LOXL1 | 9.506173 | 0.022891 | 0.719718 |
| CMBL | 0.208729 | 0.023187 | 0.726909 |
| AEBP1 | 4.510954 | 0.023341 | 0.729631 |
| RGS2 | 4.760234 | 0.02363 | 0.73359 |
| KCNK5 | 0.148689 | 0.023677 | 0.73359 |
| PKLR | 0.204651 | 0.023742 | 0.73359 |
| KRT80 | 11.54321 | 0.023784 | 0.73359 |
| ULK4 | 7.550617 | 0.023957 | 0.73359 |
| SCRN1 | 6.140212 | 0.023977 | 0.73359 |
| VSIG2 | 17.38272 | 0.02399 | 0.73359 |
| LOC388610 | 5.748971 | 0.024009 | 0.73359 |
| SPTBN2 | 0.19839 | 0.02411 | 0.734604 |
| TTPA | 0.188987 | 0.024716 | 0.750594 |
| CD7 | 11.40741 | 0.024827 | 0.750594 |
| SOD3 | 5.65966 | 0.024842 | 0.750594 |
| CES1 | 0.237307 | 0.025111 | 0.756615 |
| LYNX1 | 0.205275 | 0.025207 | 0.757394 |
| DACT2 | 12.54815 | 0.025322 | 0.758746 |
| CLGN | 0.12908 | 0.025494 | 0.760414 |
| CCL21 | 4.541235 | 0.025606 | 0.760414 |
| KLB | 0.174908 | 0.025629 | 0.760414 |
| PKHD1 | 5.727669 | 0.025658 | 0.760414 |
| SLC1A3 | 7.834758 | 0.025751 | 0.7611 |
| EREG | 22.40741 | 0.025846 | 0.761814 |
| SH2D3A | 12.38519 | 0.026531 | 0.779897 |
| GJA1 | 6.212963 | 0.027125 | 0.793789 |
| ASPN | 5.139601 | 0.02715 | 0.793789 |
| MSC | 7.709402 | 0.027309 | 0.796295 |
| SOX4 | 5.104123 | 0.027488 | 0.798692 |
| WNT4 | 22 | 0.027539 | 0.798692 |
| HLA-DMB | 4.718346 | 0.027684 | 0.800756 |
| CD83 | 5.335097 | 0.027789 | 0.801666 |
| TBX15 | 0.144215 | 0.028066 | 0.805879 |
| C8orf80 | 0.187745 | 0.028084 | 0.805879 |
| TAX1BP3 | 4.809671 | 0.028293 | 0.807789 |
| PLVAP | 4.614512 | 0.028299 | 0.807789 |
| HLA-DRB1 | 4.201028 | 0.028389 | 0.808228 |
| HBG1 | 36.66667 | 0.028679 | 0.81434 |
| LBP | 0.248155 | 0.029103 | 0.822358 |
| MRO | 0.075446 | 0.029113 | 0.822358 |
| C4BPB | 0.244622 | 0.029266 | 0.823471 |
| RAB25 | 21.59259 | 0.029354 | 0.823471 |
| PMP22 | 5.01188 | 0.029402 | 0.823471 |
| CCL8 | 10.8642 | 0.029533 | 0.823471 |
| GSTP1 | 4.229599 | 0.029576 | 0.823471 |
| CCL5 | 5.923077 | 0.029608 | 0.823471 |
| MOCOS | 0.210979 | 0.029745 | 0.823905 |
| FAM148B | 6.222222 | 0.029775 | 0.823905 |
| DUSP8 | 6.128824 | 0.0299 | 0.825261 |
| C15orf48 | 16.2963 | 0.030057 | 0.827487 |
| SLC23A1 | 0.159144 | 0.030417 | 0.834782 |
| CDC42EP5 | 8.781893 | 0.030476 | 0.834782 |
| HLA-DMA | 4.547192 | 0.030587 | 0.835716 |
| HAS1 | 9.89418 | 0.030885 | 0.841737 |
| FMO2 | 21.18519 | 0.0313 | 0.850908 |
| FLNC | 6.824074 | 0.031672 | 0.858864 |
| F2RL1 | 5.354497 | 0.031995 | 0.865462 |
| IGF1 | 0.207462 | 0.032103 | 0.866234 |
| ANXA1 | 4.332745 | 0.032318 | 0.869858 |
| MAT1A | 0.255882 | 0.032454 | 0.870339 |
| CYGB | 5.449074 | 0.032577 | 0.870339 |
| AKR1C4 | 0.235867 | 0.032684 | 0.870339 |
| HIG2 | 6.473251 | 0.032854 | 0.870339 |
| TSPAN33 | 0.216786 | 0.032857 | 0.870339 |
| LRIG1 | 0.22335 | 0.032872 | 0.870339 |
| CYP51A1 | 0.235765 | 0.032897 | 0.870339 |
| HOPX | 13.24074 | 0.033076 | 0.872945 |
| PRAGMIN | 5.951691 | 0.033218 | 0.874436 |
| CPZ | 7.537037 | 0.033294 | 0.874436 |
| PLIN | 0.107843 | 0.033511 | 0.878002 |
| GPX2 | 0.253305 | 0.033624 | 0.878845 |
| ST3GAL1 | 0.227839 | 0.033978 | 0.885965 |
| FDPS | 0.243228 | 0.034226 | 0.886485 |
| ATF5 | 0.259578 | 0.034231 | 0.886485 |
| YPEL2 | 0.231041 | 0.034243 | 0.886485 |
| DUSP1 | 3.890112 | 0.03441 | 0.888678 |
| C11orf9 | 4.37648 | 0.034521 | 0.889426 |
| C21orf63 | 5.962963 | 0.034665 | 0.891022 |
| GPR125 | 0.234641 | 0.034772 | 0.891656 |
| SLC22A1 | 0.257278 | 0.03518 | 0.900005 |
| EMP1 | 3.957997 | 0.03529 | 0.900679 |
| SLCO2A1 | 6.111111 | 0.035454 | 0.900849 |
| PLAU | 5.362007 | 0.035537 | 0.900849 |
| CYP26A1 | 0.058201 | 0.035604 | 0.900849 |
| C6orf142 | 0.141707 | 0.035629 | 0.900849 |
| SNAI1 | 6.925926 | 0.035949 | 0.906844 |
| COL6A3 | 3.997892 | 0.036214 | 0.911391 |
| NSDHL | 0.228681 | 0.036601 | 0.917138 |
| NQO1 | 7.333333 | 0.036611 | 0.917138 |
| AGPAT9 | 4.835749 | 0.036992 | 0.924549 |
| ANXA2 | 3.875876 | 0.037905 | 0.938873 |
| SELENBP1 | 0.260495 | 0.037947 | 0.938873 |
| LARP6 | 9.312169 | 0.038001 | 0.938873 |
| C20orf77 | 0.24245 | 0.038077 | 0.938873 |
| SC5DL | 0.249701 | 0.038108 | 0.938873 |
| MT1M | 0.255982 | 0.038111 | 0.938873 |
| PDGFD | 6.62716 | 0.038181 | 0.938873 |
| CD24 | 4.139785 | 0.038328 | 0.938873 |
| ARL4C | 4.15624 | 0.038344 | 0.938873 |
| COL15A1 | 7.822222 | 0.038464 | 0.939674 |
| MMP19 | 4.159098 | 0.038732 | 0.944093 |
| ARG2 | 5.104575 | 0.038896 | 0.945979 |
| SEC11C | 0.256226 | 0.039134 | 0.949638 |
| PHGDH | 0.249148 | 0.039321 | 0.952053 |
| TMSB10 | 3.735298 | 0.039524 | 0.954821 |
| FCN3 | 0.26079 | 0.039643 | 0.955565 |
| 7A5 | 14.93827 | 0.040116 | 0.963123 |
| FOXA1 | 0.214616 | 0.040218 | 0.963123 |
| SULT1C4 | 9.91358 | 0.040311 | 0.963123 |
| GZMB | 9.91358 | 0.040311 | 0.963123 |
| CHST4 | 5.703704 | 0.040471 | 0.964808 |
| CYBRD1 | 4.306878 | 0.040715 | 0.968507 |
| NR1D1 | 4.265203 | 0.041101 | 0.974244 |
| HBEGF | 4.395062 | 0.041136 | 0.974244 |
| HSPA6 | 9.079365 | 0.041337 | 0.976862 |
| MDK | 4.242999 | 0.041619 | 0.981397 |
| COBLL1 | 0.251469 | 0.041711 | 0.981437 |
| SORD | 0.258479 | 0.041984 | 0.985252 |
| BHMT | 0.271434 | 0.042058 | 0.985252 |
| HLA-A | 3.686936 | 0.042158 | 0.985252 |
| PEG3 | 0.167532 | 0.042307 | 0.985252 |
| UGT1A6 | 0.132132 | 0.042353 | 0.985252 |
| TCEA2 | 4.089448 | 0.042418 | 0.985252 |
| ANPEP | 0.273536 | 0.042662 | 0.9888 |
| MXRA8 | 4.306878 | 0.042772 | 0.989222 |
| GABRP | 19.14815 | 0.043399 | 1 |
| DUSP2 | 6.087146 | 0.043571 | 1 |
| NUCB2 | 0.25697 | 0.043707 | 1 |
| ENO2 | 7.876543 | 0.044126 | 1 |
| SLC46A1 | 0.199761 | 0.044202 | 1 |
| AGL | 0.233618 | 0.044286 | 1 |
| OASL | 4.690154 | 0.044831 | 1 |
| SLC29A4 | 0.167756 | 0.044878 | 1 |
| CES3 | 0.189919 | 0.044922 | 1 |
| NFKBIE | 4.656085 | 0.045145 | 1 |
| CCND2 | 4.820988 | 0.045179 | 1 |
| KLF5 | 5.789474 | 0.045275 | 1 |
| ST14 | 3.998628 | 0.045568 | 1 |
| HMGCR | 0.252628 | 0.045691 | 1 |
| MAMDC4 | 0.206219 | 0.046182 | 1 |
| TMEM97 | 0.258025 | 0.046299 | 1 |
| LMAN1 | 0.265585 | 0.046443 | 1 |
| CD34 | 4.585702 | 0.046748 | 1 |
| PCDH24 | 0.253669 | 0.047591 | 1 |
| SERPINA1 | 0.286266 | 0.047934 | 1 |
| TSPAN8 | 5.214815 | 0.048063 | 1 |
| SEMA3B | 5.581481 | 0.04831 | 1 |
| SLC16A3 | 4.155556 | 0.048421 | 1 |
| RAB27A | 0.24496 | 0.048449 | 1 |
| ETNK2 | 0.278408 | 0.048632 | 1 |
| IGJ | 4.653021 | 0.048823 | 1 |
| PSKH1 | 0.236038 | 0.048955 | 1 |
| MICAL1 | 4.617284 | 0.04912 | 1 |
| CLDN15 | 5.748971 | 0.049287 | 1 |
| ACHE | 8.046296 | 0.04934 | 1 |
| LOC388503 | 0.280709 | 0.049826 | 1 |

**Supplementary Table 7. List of genes mapped from the common SNPs in the whole exome data and the internal list of GWAS**

| **EntrezID** | **Gene Symbol** | **Gene Description** |
| --- | --- | --- |
| 368 | ABCC6 | ATP-binding cassette, sub-family C, member 6 pseudogene 2; ATP-binding cassette, sub-family C (CFTR/MRP), member 6 |
| 593 | BCKDHA | branched chain keto acid dehydrogenase E1, alpha polypeptide |
| 960 | CD44 | CD44 molecule (Indian blood group) |
| 1072 | CFL1 | cofilin 1 (non-muscle) |
| 1179 | CLCA1 | chloride channel accessory 1 |
| 1187 | CLCNKA | chloride channel Ka |
| 1292 | COL6A2 | collagen, type VI, alpha 2 |
| 1297 | COL9A1 | collagen, type IX, alpha 1 |
| 1629 | DBT | dihydrolipoamide branched chain transacylase E2 |
| 1994 | ELAVL1 | ELAV (embryonic lethal, abnormal vision, Drosophila)-like 1 (Hu antigen R) |
| 2195 | Fat1 | FAT tumor suppressor homolog 1 (Drosophila) |
| 2203 | Fbp1 | fructose-1,6-bisphosphatase 1 |
| 2317 | FLNB | filamin B, beta (actin binding protein 278) |
| 2444 | FRK | fyn-related kinase |
| 2524 | FUT2 | fucosyltransferase 2 (secretor status included) |
| 2638 | gc | group-specific component (vitamin D binding protein) |
| 2868 | GRK4 | G protein-coupled receptor kinase 4 |
| 3046 | HBE1 | hemoglobin, epsilon 1 |
| 3048 | HBG2 | hemoglobin, gamma G |
| 3064 | HTT | huntingtin |
| 3371 | TNC | tenascin C |
| 3373 | Hyal1 | hyaluronoglucosaminidase 1 |
| 3484 | IGFBP1 | insulin-like growth factor binding protein 1 |
| 3508 | IGHMBP2 | immunoglobulin mu binding protein 2 |
| 3601 | IL15RA | interleukin 15 receptor, alpha |
| 3823 | Klrc3 | killer cell lectin-like receptor subfamily C, member 3 |
| 3882 | Krt32 | keratin 32 |
| 4008 | Lmo7 | LIM domain 7 |
| 4036 | LRP2 | low density lipoprotein-related protein 2 |
| 4240 | MFGE8 | milk fat globule-EGF factor 8 protein |
| 4259 | Mgst3 | microsomal glutathione S-transferase 3 |
| 4522 | mthfd1 | methylenetetrahydrofolate dehydrogenase (NADP+ dependent) 1, methenyltetrahydrofolate cyclohydrolase, formyltetrahydrofolate synthetase |
| 4585 | MUC4 | mucin 4, cell surface associated |
| 4760 | NEUROD1 | neurogenic differentiation 1 |
| 4892 | Nrap | nebulin-related anchoring protein |
| 4992 | OR1F1 | olfactory receptor, family 1, subfamily F, member 1 |
| 5002 | Slc22a18 | solute carrier family 22, member 18 |
| 5003 | SLC22A18AS | solute carrier family 22 (organic cation transporter), member 18 antisense |
| 5108 | pcm1 | pericentriolar material 1 |
| 5176 | Serpinf1 | serpin peptidase inhibitor, clade F (alpha-2 antiplasmin, pigment epithelium derived factor), member 1 |
| 5275 | Serpinb13 | serpin peptidase inhibitor, clade B (ovalbumin), member 13 |
| 5288 | PIK3C2G | phosphoinositide-3-kinase, class 2, gamma polypeptide |
| 5625 | PRODH | proline dehydrogenase (oxidase) 1 |
| 5858 | PZP | pregnancy-zone protein |
| 6332 | SCN7A | sodium channel, voltage-gated, type VII, alpha |
| 6370 | ccl25 | chemokine (C-C motif) ligand 25 |
| 6491 | STIL | SCL/TAL1 interrupting locus |
| 6493 | SIM2 | single-minded homolog 2 (Drosophila) |
| 6519 | Slc3a1 | solute carrier family 3 (cystine, dibasic and neutral amino acid transporters, activator of cystine, dibasic and neutral amino acid transport), member 1 |
| 6565 | Slc15a2 | solute carrier family 15 (H+/peptide transporter), member 2 |
| 6585 | slit1 | slit homolog 1 (Drosophila) |
| 6614 | SIGLEC1 | sialic acid binding Ig-like lectin 1, sialoadhesin |
| 6653 | SORL1 | sortilin-related receptor, L(DLR class) A repeats-containing |
| 7143 | TNR | tenascin R (restrictin, janusin) |
| 7766 | ZNF223 | zinc finger protein 223 |
| 7772 | ZNF229 | zinc finger protein 229 |
| 7866 | Ifrd2 | interferon-related developmental regulator 2 |
| 8029 | cubn | cubilin (intrinsic factor-cobalamin receptor) |
| 8100 | IFT88 | intraflagellar transport 88 homolog (Chlamydomonas) |
| 8214 | DGCR6 | DiGeorge syndrome critical region gene 6 |
| 8302 | KLRC4 | killer cell lectin-like receptor subfamily C, member 4 |
| 8372 | HYAL3 | hyaluronoglucosaminidase 3 |
| 8558 | cdk10 | cyclin-dependent kinase 10 |
| 8602 | NOP14 | NOP14 nucleolar protein homolog (yeast) |
| 8701 | DNAH11 | dynein, axonemal, heavy chain 11 |
| 8735 | MYH13 | myosin, heavy chain 13, skeletal muscle |
| 8736 | myom1 | myomesin 1, 185kDa |
| 8793 | TNFRSF10D | tumor necrosis factor receptor superfamily, member 10d, decoy with truncated death domain |
| 8871 | SYNJ2 | synaptojanin 2 |
| 8877 | SPHK1 | sphingosine kinase 1 |
| 8899 | prpf4b | similar to hCG1820375; PRP4 pre-mRNA processing factor 4 homolog B (yeast) |
| 9013 | TAF1C | TATA box binding protein (TBP)-associated factor, RNA polymerase I, C, 110kDa |
| 9154 | Slc28a1 | solute carrier family 28 (sodium-coupled nucleoside transporter), member 1 |
| 9389 | SLC22A14 | solute carrier family 22, member 14 |
| 9510 | Adamts1 | ADAM metallopeptidase with thrombospondin type 1 motif, 1 |
| 9518 | Gdf15 | growth differentiation factor 15 |
| 9581 | PREPL | prolyl endopeptidase-like |
| 9609 | rab36 | RAB36, member RAS oncogene family |
| 9808 | KIAA0087 | KIAA0087 |
| 10160 | FARP1 | FERM, RhoGEF (ARHGEF) and pleckstrin domain protein 1 (chondrocyte-derived) |
| 10345 | TRDN | triadin |
| 10350 | Abca9 | ATP-binding cassette, sub-family A (ABC1), member 9 |
| 10400 | pemt | phosphatidylethanolamine N-methyltransferase |
| 10531 | PITRM1 | pitrilysin metallopeptidase 1 |
| 10616 | RBCK1 | RanBP-type and C3HC4-type zinc finger containing 1 |
| 10827 | Fam114a2 | family with sequence similarity 114, member A2 |
| 11066 | snrnp35 | ATP-binding cassette, sub-family B (MDR/TAP), member 5; small nuclear ribonucleoprotein 35kDa (U11/U12) |
| 11148 | HHLA2 | HERV-H LTR-associating 2 |
| 11196 | SEC23IP | SEC23 interacting protein |
| 11201 | poli | polymerase (DNA directed) iota |
| 11214 | AKAP13 | A kinase (PRKA) anchor protein 13 |
| 11264 | PXMP4 | peroxisomal membrane protein 4, 24kDa |
| 22824 | Hspa4l | heat shock 70kDa protein 4-like |
| 22838 | RNF44 | ring finger protein 44 |
| 22876 | INPP5F | inositol polyphosphate-5-phosphatase F |
| 23013 | spen | spen homolog, transcriptional regulator (Drosophila) |
| 23217 | ZFR2 | zinc finger RNA binding protein 2 |
| 23223 | Rrp12 | ribosomal RNA processing 12 homolog (S. cerevisiae) |
| 23224 | SYNE2 | spectrin repeat containing, nuclear envelope 2 |
| 23279 | NUP160 | nucleoporin 160kDa |
| 23325 | KIAA1033 | KIAA1033 |
| 23345 | SYNE1 | spectrin repeat containing, nuclear envelope 1 |
| 23351 | khnyn | KIAA0323 |
| 23362 | PSD3 | pleckstrin and Sec7 domain containing 3 |
| 23460 | Abca6 | ATP-binding cassette, sub-family A (ABC1), member 6 |
| 23627 | Prnd | prion protein 2 (dublet) |
| 23767 | Flrt3 | fibronectin leucine rich transmembrane protein 3 |
| 24142 | NAT6 | N-acetyltransferase 6 (GCN5-related) |
| 25878 | MXRA5 | matrix-remodelling associated 5 |
| 25938 | heatr5a | HEAT repeat containing 5A |
| 27122 | DKK3 | dickkopf homolog 3 (Xenopus laevis) |
| 27130 | Invs | inversin |
| 27283 | TINAG | tubulointerstitial nephritis antigen |
| 28671 | TRAV13-1 | T cell receptor alpha variable 13-1 |
| 29070 | CCDC113 | coiled-coil domain containing 113 |
| 29119 | CTNNA3 | catenin (cadherin-associated protein), alpha 3 |
| 50617 | ATP6V0A4 | ATPase, H+ transporting, lysosomal V0 subunit a4 |
| 50999 | Tmed5 | transmembrane emp24 protein transport domain containing 5 |
| 51222 | ZNF219 | zinc finger protein 219 |
| 51321 | Amz2 | archaelysin family metallopeptidase 2 |
| 51473 | DCDC2 | doublecortin domain containing 2 |
| 51530 | zc3hc1 | zinc finger, C3HC-type containing 1 |
| 51700 | cyb5r2 | cytochrome b5 reductase 2 |
| 53827 | FXYD5 | FXYD domain containing ion transport regulator 5 |
| 53904 | MYO3A | myosin IIIA |
| 54465 | etaa1 | Ewing tumor-associated antigen 1 |
| 54502 | RBM47 | RNA binding motif protein 47 |
| 54522 | ankrd16 | ankyrin repeat domain 16 |
| 54596 | L1TD1 | LINE-1 type transposase domain containing 1 |
| 54714 | CNGB3 | cyclic nucleotide gated channel beta 3 |
| 54860 | Ms4a12 | membrane-spanning 4-domains, subfamily A, member 12 |
| 54881 | Tex10 | testis expressed 10 |
| 55062 | WIPI1 | WD repeat domain, phosphoinositide interacting 1 |
| 55101 | ATP5SL | ATP5S-like |
| 55106 | SLFN12 | schlafen family member 12 |
| 55132 | Larp1b | La ribonucleoprotein domain family, member 1B |
| 55258 | Thnsl2 | threonine synthase-like 2 (S. cerevisiae) |
| 55584 | CHRNA9 | cholinergic receptor, nicotinic, alpha 9 |
| 55614 | KIF16B | kinesin family member 16B |
| 55624 | POMGNT1 | protein O-linked mannose beta1,2-N-acetylglucosaminyltransferase |
| 55742 | PARVA | parvin, alpha |
| 55757 | Uggt2 | UDP-glucose ceramide glucosyltransferase-like 2 |
| 55781 | RIOK2 | RIO kinase 2 (yeast) |
| 55833 | UBAP2 | ubiquitin associated protein 2 |
| 56547 | MMP26 | matrix metallopeptidase 26 |
| 56890 | Mdm1 | Mdm1 nuclear protein homolog (mouse) |
| 56893 | UBQLN4 | ubiquilin 4 |
| 56916 | Smarcad1 | SWI/SNF-related, matrix-associated actin-dependent regulator of chromatin, subfamily a, containing DEAD/H box 1 |
| 57127 | RHBG | Rh family, B glycoprotein (gene/pseudogene) |
| 57188 | Adamtsl3 | ADAMTS-like 3 |
| 57539 | WDR35 | WD repeat domain 35 |
| 57572 | Dock6 | dedicator of cytokinesis 6 |
| 57647 | DHX37 | DEAH (Asp-Glu-Ala-His) box polypeptide 37 |
| 58499 | znf462 | zinc finger protein 462 |
| 60401 | EDA2R | ectodysplasin A2 receptor |
| 60681 | Fkbp10 | FK506 binding protein 10, 65 kDa |
| 63893 | UBE2O | ubiquitin-conjugating enzyme E2O |
| 64651 | CSRNP1 | cysteine-serine-rich nuclear protein 1 |
| 79345 | OR51B2 | olfactory receptor, family 51, subfamily B, member 2 |
| 79443 | Fyco1 | FYVE and coiled-coil domain containing 1 |
| 79482 | OR5AL1 | olfactory receptor, family 5, subfamily AL, member 1 (gene/pseudogene) |
| 79671 | NLRX1 | NLR family member X1 |
| 79677 | SMC6 | structural maintenance of chromosomes 6 |
| 79785 | RERGL | RERG/RAS-like |
| 79841 | agbl2 | ATP/GTP binding protein-like 2 |
| 79849 | PDZD3 | PDZ domain containing 3 |
| 80010 | Rmi1 | RMI1, RecQ mediated genome instability 1, homolog (S. cerevisiae) |
| 80144 | FRAS1 | Fraser syndrome 1 |
| 80198 | MUS81 | MUS81 endonuclease homolog (S. cerevisiae) |
| 80205 | CHD9 | chromodomain helicase DNA binding protein 9 |
| 80274 | Scube1 | signal peptide, CUB domain, EGF-like 1 |
| 80309 | SPHKAP | SPHK1 interactor, AKAP domain containing |
| 81618 | ITM2C | integral membrane protein 2C |
| 81704 | DOCK8 | dedicator of cytokinesis 8 |
| 83878 | Ushbp1 | Usher syndrome 1C binding protein 1 |
| 84224 | NBPF3 | neuroblastoma breakpoint family, member 3 |
| 84467 | FBN3 | fibrillin 3 |
| 84639 | IL1F10 | interleukin 1 family, member 10 (theta) |
| 84700 | MYO18B | myosin XVIIIB |
| 84899 | Tmtc4 | transmembrane and tetratricopeptide repeat containing 4 |
| 90075 | ZNF30 | zinc finger protein 30 |
| 90313 | TP53I13 | tumor protein p53 inducible protein 13 |
| 90668 | LRRC16B | leucine rich repeat containing 16B |
| 91373 | Uap1l1 | UDP-N-acteylglucosamine pyrophosphorylase 1-like 1 |
| 91862 | marveld3 | MARVEL domain containing 3 |
| 91937 | TIMD4 | T-cell immunoglobulin and mucin domain containing 4 |
| 92106 | oxnad1 | oxidoreductase NAD-binding domain containing 1 |
| 92196 | DAPL1 | death associated protein-like 1 |
| 93190 | C1orf158 | chromosome 1 open reading frame 158 |
| 113146 | AHNAK2 | AHNAK nucleoprotein 2 |
| 114780 | PKD1L2 | polycystic kidney disease 1-like 2 |
| 114784 | CSMD2 | CUB and Sushi multiple domains 2 |
| 114826 | SMYD4 | SET and MYND domain containing 4 |
| 116236 | ABHD15 | abhydrolase domain containing 15 |
| 119679 | OR52J3 | olfactory receptor, family 52, subfamily J, member 3 |
| 119692 | OR51S1 | olfactory receptor, family 51, subfamily S, member 1 |
| 122618 | PLD4 | phospholipase D family, member 4 |
| 124044 | SPATA2L | spermatogenesis associated 2-like |
| 125965 | Cox6b2 | cytochrome c oxidase subunit VIb polypeptide 2 (testis) |
| 126364 | LRRC25 | leucine rich repeat containing 25 |
| 126370 | OR1I1 | olfactory receptor, family 1, subfamily I, member 1 |
| 126375 | ZNF792 | zinc finger protein 792 |
| 126549 | Ankle1 | ankyrin repeat and LEM domain containing 1 |
| 126767 | AADACL3 | arylacetamide deacetylase-like 3 |
| 127602 | DNAH14 | dynein, axonemal, heavy chain 14 |
| 128372 | OR6N1 | olfactory receptor, family 6, subfamily N, member 1 |
| 129025 | ZNF280A | zinc finger protein 280A |
| 136288 | C7orf57 | chromosome 7 open reading frame 57 |
| 138881 | OR1L8 | olfactory receptor, family 1, subfamily L, member 8 |
| 138882 | OR1N2 | olfactory receptor, family 1, subfamily N, member 2 |
| 140733 | macrod2 | MACRO domain containing 2 |
| 146723 | C17orf77 | chromosome 17 open reading frame 77 |
| 147929 | ZNF565 | zinc finger protein 565 |
| 152137 | CCDC50 | coiled-coil domain containing 50 |
| 159989 | CCDC67 | coiled-coil domain containing 67 |
| 162972 | ZNF550 | zinc finger protein 550 |
| 163786 | Sass6 | spindle assembly 6 homolog (C. elegans) |
| 169611 | OLFML2A | olfactomedin-like 2A |
| 199223 | ttc21a | tetratricopeptide repeat domain 21A |
| 202243 | CCDC125 | coiled-coil domain containing 125 |
| 202500 | TCTE1 | t-complex-associated-testis-expressed 1 |
| 203427 | Slc25a43 | solute carrier family 25, member 43 |
| 219479 | OR5R1 | olfactory receptor, family 5, subfamily R, member 1 |
| 219790 | RTKN2 | rhotekin 2 |
| 220323 | OAF | OAF homolog (Drosophila) |
| 221074 | SLC39A12 | solute carrier family 39 (zinc transporter), member 12 |
| 221806 | VWDE | von Willebrand factor D and EGF domains |
| 221935 | SDK1 | sidekick homolog 1, cell adhesion molecule (chicken); hypothetical LOC730351 |
| 245802 | MS4A6E | membrane-spanning 4-domains, subfamily A, member 6E |
| 254122 | SNX32 | sorting nexin 32 |
| 255189 | PLA2G4F | phospholipase A2, group IVF |
| 255239 | ANKK1 | ankyrin repeat and kinase domain containing 1 |
| 255394 | TCP11L2 | t-complex 11 (mouse)-like 2 |
| 256051 | ZNF549 | zinc finger protein 549 |
| 256297 | PTF1A | pancreas specific transcription factor, 1a |
| 266747 | RGL4 | ral guanine nucleotide dissociation stimulator-like 4 |
| 282763 | OR51B5 | olfactory receptor, family 51, subfamily B, member 5 |
| 283152 | CCDC153 | coiled-coil domain containing 153 |
| 284018 | C17orf58 | chromosome 17 open reading frame 58 |
| 284415 | VSTM1 | V-set and transmembrane domain containing 1 |
| 284418 | Fam71e2 | family with sequence similarity 71, member E2 |
| 284958 | nt5dc4 | 5'-nucleotidase domain containing 4 |
| 285315 | c3orf33 | chromosome 3 open reading frame 33 |
| 338751 | OR52L1 | olfactory receptor, family 52, subfamily L, member 1 |
| 340273 | Abcb5 | ATP-binding cassette, sub-family B (MDR/TAP), member 5; small nuclear ribonucleoprotein 35kDa (U11/U12) |
| 341568 | OR8S1 | olfactory receptor, family 8, subfamily S, member 1 |
| 344561 | GPR148 | G protein-coupled receptor 148 |
| 348654 | GEN1 | Gen homolog 1, endonuclease (Drosophila) |
| 374308 | PTCHD3 | patched domain containing 3 |
| 374907 | B3GNT8 | UDP-GlcNAc:betaGal beta-1,3-N-acetylglucosaminyltransferase 8 |
| 375298 | CERKL | ceramide kinase-like |
| 390157 | OR8K1 | olfactory receptor, family 8, subfamily K, member 1 |
| 390882 | OR7G2 | olfactory receptor, family 7, subfamily G, member 2 |
| 391196 | OR2M7 | olfactory receptor, family 2, subfamily M, member 7 |
| 399814 | C10orf120 | chromosome 10 open reading frame 120 |
| 403284 | OR6C68 | olfactory receptor, family 6, subfamily C, member 68 |
| 440193 | CCDC88C | coiled-coil domain containing 88C |
| 440822 | PIWIL3 | piwi-like 3 (Drosophila) |
| 441151 | TMEM151B | transmembrane protein 151B |
| 643866 | Cbln3 | cerebellin 3 precursor |
| 1E+08 | SNORD121B | small nucleolar RNA, C/D box 121B |
| 1E+08 | CD300LD | CD300 molecule-like family member d |

**Supplementary Table 8. TDT results for the SNPs near differentially regulated genes (“TDT_GWAS” of BA_SYSBIO_SUPP.xlsx)**

**Supplementary Table 9. List of enriched Gene Ontology (GO) terms in the whole exome network (“GO_terms_wholeexomenetwork” of BA_SYSBIO_SUPP.xlsx)**
